# Copper-catalyzed dehydrogenative borylation of terminal alkynes with pinacolborane†
†Electronic supplementary information (ESI) available. See DOI: 10.1039/c6sc02668k



**DOI:** 10.1039/c6sc02668k

**Published:** 2016-08-09

**Authors:** Erik A. Romero, Rodolphe Jazzar, Guy Bertrand

**Affiliations:** a UCSD-CNRS Joint Research Chemistry Laboratory (UMI 3555) , Department of Chemistry and Biochemistry , University of California San Diego , La Jolla , CA 92093-0358 , USA . Email: guybertrand@ucsd.edu

## Abstract

LCuOTf complexes [L = cyclic (alkyl)(amino)carbenes (CAACs) or N-heterocyclic carbenes (NHCs)] selectively promote the dehydrogenative borylation of C(sp)–H bonds at room temperature. It is shown that σ,π-bis(copper) acetylide and copper hydride complexes are the key catalytic species.

## Abstract 2

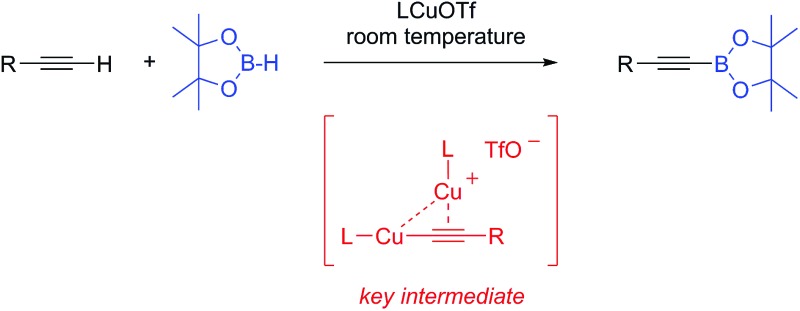
LCuOTf complexes (L = CAACs or NHCs) selectively promote the dehydrogenative borylation of C(sp)–H bonds at room temperature.

Popularized by the Suzuki–Miyaura reaction, organoboronic esters and acids are now regarded as key building blocks for compounds with applications ranging from material to life sciences. Consequently, numerous methodologies have been developed to access these valuable substrates.^1^ Following ground-breaking reports by Hartwig *et al.*,^2^ and Smith *et al.*,^3^ the catalytic dehydrogenative borylation of C(sp^3^)–H bonds and C(sp^2^)–H bonds are now well documented.^4^ In contrast, for C(sp)–H bonds there are only a few reports by Ozerov *et al.* ^5^ using iridium and palladium complexes supported by pincer ligands, and one by Tsuchimoto *et al.* ^6^ with zinc triflate, which is only effective with 1,8-naphthalenediaminatoborane as the boron partner. One of the difficulties of the dehydrogenative borylation of terminal alkynes is the competing hydroboration of the triple bond, which affords alkenyl boronic esters.^7^

We recently reported^8^ on the role of the X ligand in the mechanism of the LCuX catalyzed azide-alkyne cycloaddition (CuAAC reaction) [L = cyclic (alkyl)(amino)carbene].^9,10^ Herein we show that these results allow for the rational design of copper catalysts that selectively promote the dehydrogenative borylation of terminal alkynes with pinacolborane.

In the above mentioned study, we found that in the presence of triethylamine, LCuOTf reacts with terminal alkynes to give the catalytically active σ,π-bis(copper) acetylides **A**,^8,11^ along with ammonium triflate (Scheme 1). We hypothesized that in dinuclear complexes **A**, the triple bond is protected which should prevent the classical hydroboration reaction leading to alkenyl boronic esters. Instead, the highly polarized copper–carbon bond could undergo a σ-bond metathesis with pinacolborane (**TS**) to afford the desired alkynyl boronic ester **B**, as well as the copper hydride **C**. The latter should react with triethylammonium triflate to regenerate LCuOTf and triethylamine with the elimination of dihydrogen.

**Scheme 1:**
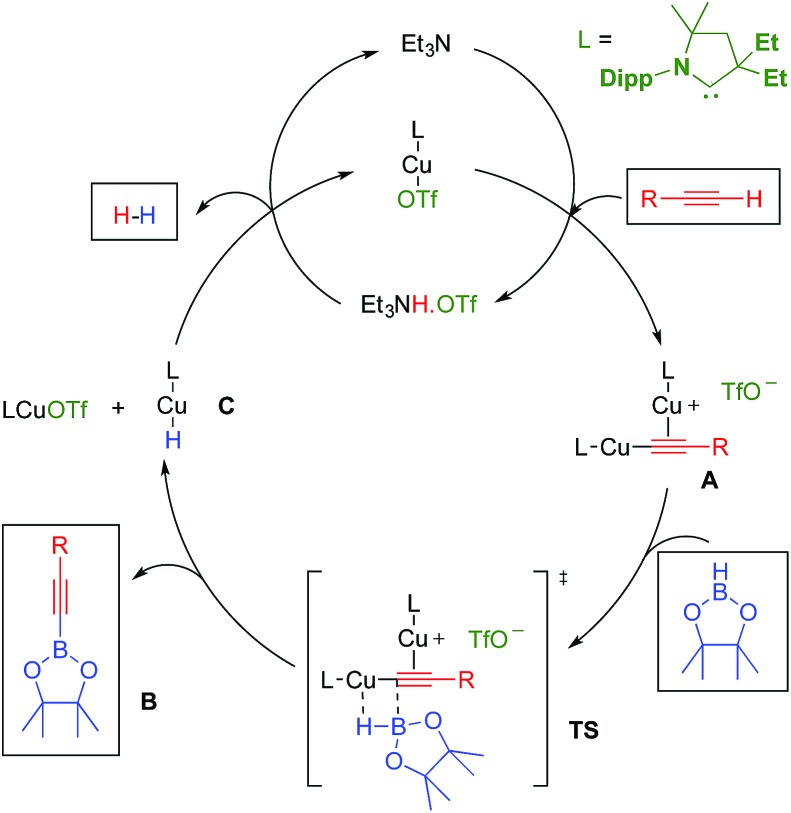
Hypothetical mechanism for the LCuOTf induced dehydrogenative borylation of terminal alkynes.

We first checked that in the absence of catalyst no reaction occurred between *p*-tolylacetylene **1a** and pinacolborane in C_6_D_6_ at room temperature for 2 hours (Table 1, entry 1). In the presence of 1 mol% of **L_1_**CuOTf, no significant reaction occurred either because of the difficulty of the triflate to deprotonate the alkyne^8^ (entry 2). In order to promote the deprotonation of the alkyne, 1 mol% of Et_3_N was added which resulted in immediate hydrogen evolution (as characterized by a singlet at 4.47 ppm in ^1^H NMR). The major product was the alkynyl boronic ester **B_1_** (48%), but the alkenyl boronic ester **D_1_** (11%), and the styrene derivative **E_1_** (7%) were also formed (entry 3).

**Table 1:** Optimization of the dehydrogenative borylation reaction^*a*^

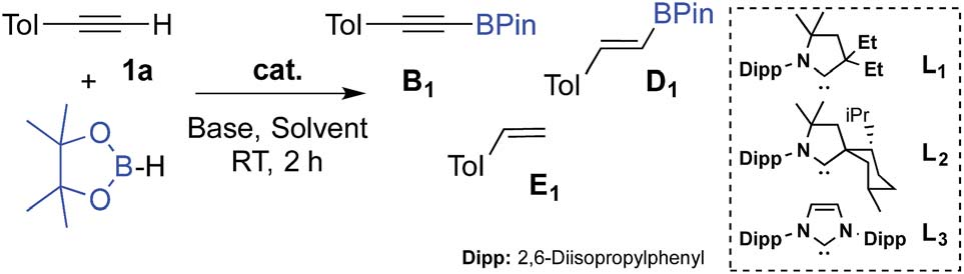								
Entry	Cat. (mol%)	Base (mol%)	Solvent	Conc. (M)	**1a** ^*b*^ (%)	**B_1_** ^*b*^ (%)	**D_1_** ^*b*^ (%)	**E_1_** ^*b*^ (%)
1	—	—	C_6_D_6_	1.4	100	0	0	0
2	L_1_CuOTf (1)	—	C_6_D_6_	1.4	86	0	6	6
3	L_1_CuOTf (1)	Et_3_N (1)	C_6_D_6_	1.4	14	48	11	7
4	CuOTf	Et_3_N (1)	C_6_D_6_	1.4	100	0	0	0
5	L_1_CuOTf (1)	Et_3_N (2)	C_6_D_6_	1.4	1	70	14	12
6	L_1_CuOTf (1)	Et_3_N (2)	CD_2_Cl_2_	1.4	12	42	6	26
7	L_1_CuOTf (1)	Et_3_N (2)	THF-d_8_	1.4	5	64	12	17
8	L_1_CuOTf (1)	Et_3_N (2)	CD_3_CN	1.4	18	38	0	6
9	L_1_CuOTf (1)	^i^PrNH_2_ (2)	C_6_D_6_	1.4	67	5	12	10
10	L_1_CuOTf (1)	^i^Pr_2_NH (2)	C_6_D_6_	1.4	47	11	13	7
11	L_1_CuOTf (1)	^i^Pr_2_NEt (2)	C_6_D_6_	1.4	10	28	45	3
12	L_1_CuOTf (1)	BnNEt_2_ (2)	C_6_D_6_	1.4	14	53	8	7
13	L_1_CuOTf (1)	DABCO (2)	C_6_D_6_	1.4	18	60	1	7
14	L_1_CuOTf (0.25)	Et_3_N (0.5)	C_6_D_6_	1.4	37	36	15	6
15	L_1_CuOTf (0.5)	Et_3_N (1)	C_6_D_6_	1.4	20	54	15	9
16	L_1_CuOTf (2.5)	Et_3_N (5)	C_6_D_6_	1.4	4	83	4	7
**17**	**L**_**1**_ **CuOTf (2.5)**	**Et**_**3**_ **N (5)**	**C**_**6**_ **D**_**6**_	**0.1**	**1**	**98**	**0**	**1**
18	L_2_CuOTf (2.5)	Et_3_N (5)	C_6_D_6_	0.1	0	96	0	4
19	L_3_CuOTf (2.5)	Et_3_N (5)	C_6_D_6_	0.1	0	92	0	8

^*a*^Reactions were carried out in a test tube for 2 h at RT under an argon atmosphere using a 1 : 1 mixture (0.69 mmol) of *p*-tolylacetylene and pinacolborane.

^*b*^Measured by NMR using 1,4-dioxane as an internal standard.

Note that no reaction occurred under ligand-free conditions (entry 4). Encouraged by these preliminary results, we further optimized the dehydrogenative borylation leading to **B_1_**. We found that a two-fold excess of Et_3_N with respect to (CAAC) CuOTf was beneficial (entry 5). By screening solvents (Entries 5–8) and base additives (Entries 9–13), we identified benzene and Et_3_N as being most appropriate for the reaction. When we increased the catalyst loading to 2.5 mol% (Entries 14–16), and decreased the concentration of the solution from 1.4 to 0.1 mol L^–1^ (entry 17) there was quantitative formation of the desired alkynyl boronic ester **B_1_** with excellent selectivity within two hours at room temperature. Notably, substitution of ligand **L_1_** for the more bulky menthyl CAAC **L_2_** or even IPr-NHC^12^ **L_3_** gave comparable results.

The scope of the dehydrogenative borylation reaction was then studied at room temperature in a benzene solution (0.1 M) using a stoichiometric mixture of alkyne and borane (1.82 mmol), 5 mol% of Et_3_N and 2.5 mol% of L_1_CuOTf (Scheme 2). This methodology is readily applicable to a broad range of terminal alkynes bearing functionalities such as OMe, CN, F, Cl, TMS and CO_2_Me. It is worth noting that electron-rich terminal alkynes require longer reaction times (12 h instead of 2 h) (**B_11–15_**). Alkynyl boronic esters **B_1–15_** were isolated in good to excellent yields *via* filtration through a short plug of dry neutral alumina using pentane as the eluent. This straightforward protocol allows for gram-scale synthesis, as shown for **B_3_**.

**Scheme 2:**
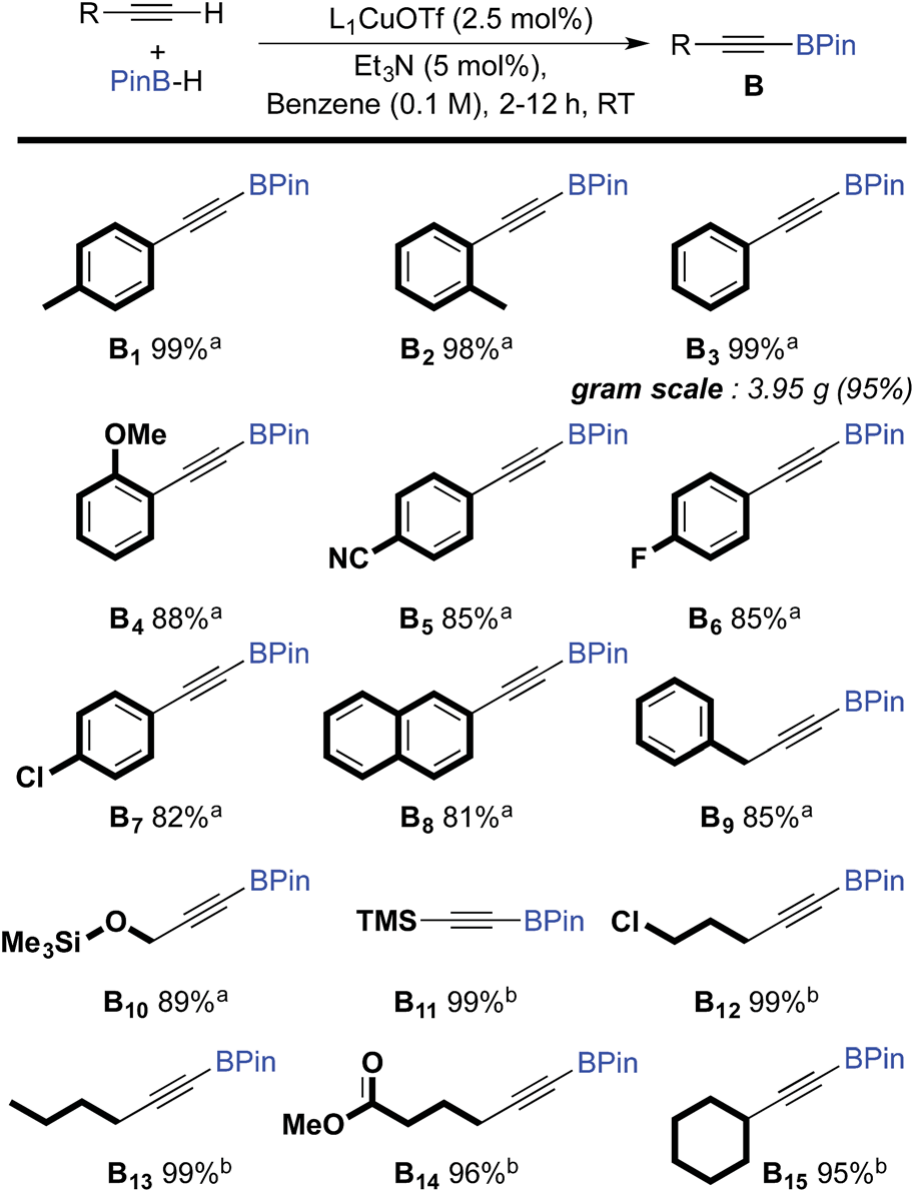
Scope of the dehydrogenative borylation of terminal alkynes. [a] Reaction time 2 h. [b] Reaction time 12 h.

With these results in hand, we performed a set of experiments in order to verify our mechanistic hypothesis. When the 2.5 mol% mononuclear complex **F** was used, with or without Et_3_N, no traces of the dehydrogenative borylation product **B_1_** were observed, instead the hydroboration product **D_1_** was quantitatively formed (Fig. 1 and Scheme 3) (see also the kinetic profile in the ESI†). In marked contrast, when 2.5 mol% Et_3_NH·OTf was added as a proton source, we observed the rapid formation of **B_1_**, and the kinetic profile was comparable with those obtained using our standard catalytic conditions (LCuOTf/Et_3_N) or the bis(copper) acetylide **A**. Since we already proved that LCuOTf/Et_3_N reacts with terminal alkynes to afford the dinuclear species **A**,^8^ we verified that similarly complex **F** reacts with Et_3_NH·OTf to give **A**. These experiments as a whole strongly suggest that the dinuclear complex **A** is pivotal in the dehydrogenative process.

**Fig. 1:**
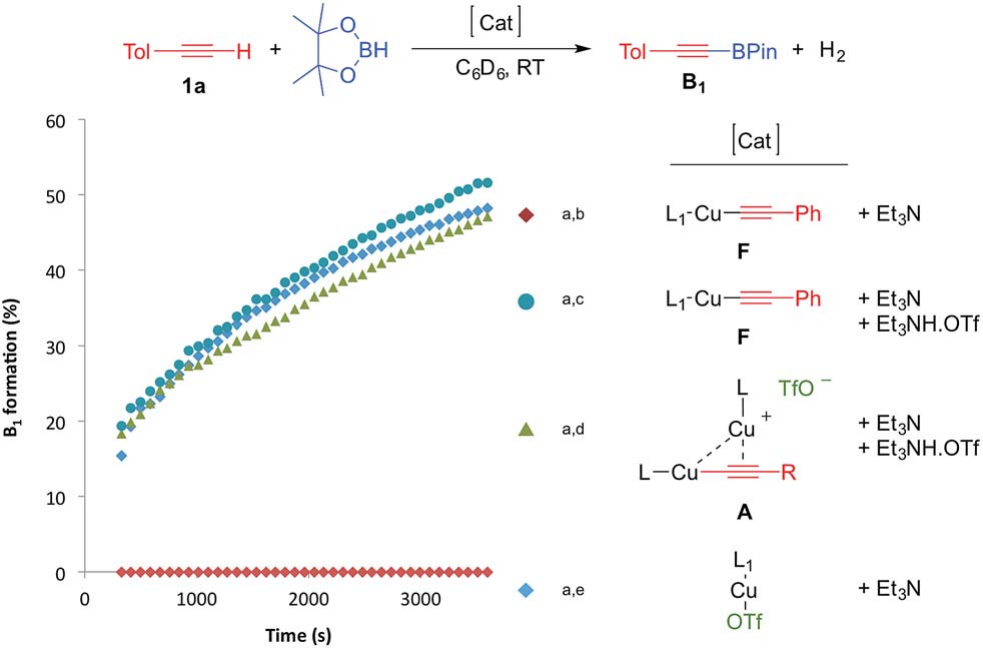
Kinetic profiles of the formation of **B_1_** using various catalytic systems. [a] Reactions were carried out in a J-Young NMR tube at RT under an argon atmosphere using a 1 : 1 mixture (0.45 mmol) of *p*-tolylacetylene and pinacolborane in 1 mL of C_6_D_6_. [b] 2.5 mol% of **F** and 5 mol% Et_3_N; no trace of **B_1_** was observed, instead **D_1_** was obtained quantitatively. [c] 2.5 mol% of **F**, Et_3_N and Et_3_NH·OTf. [d] 2.5 mol% of **A**, 3.75 mol% Et_3_N and 1.25 mol% Et_3_NH·OTf. [e] 2.5 mol% of L_1_CuOTf and 5 mol% of Et_3_N.

**Scheme 3:**
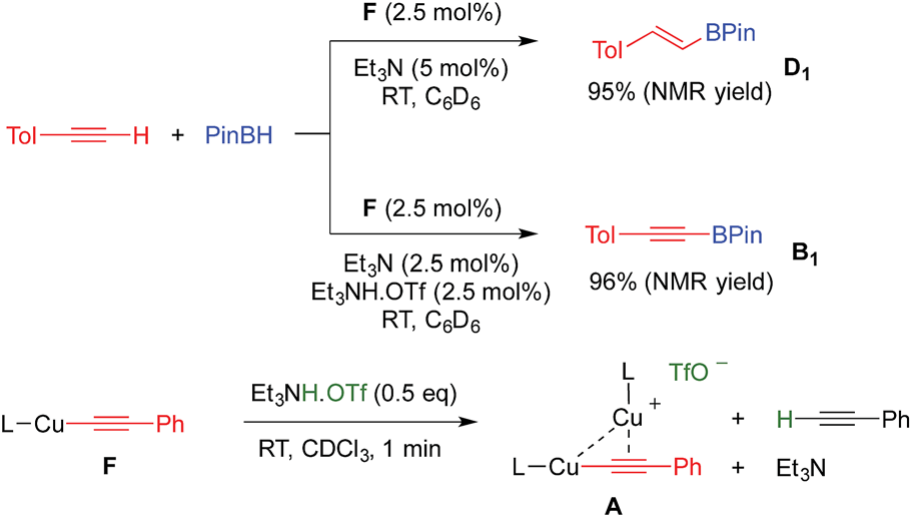
Evidence for the pivotal role of the dinuclear copper complex **A**.

The other important species in our postulated mechanism is the copper hydride **C**, a type of complex that has been proposed to play a major role in a number of catalytic transformations.^13^ While there is still no report of a monomeric mono-ligated Cu hydride,^14^ a range of dimeric species have been described by us and others.^15^ An indication of **L_1_**CuH formation in this process is the observation of a small amount of styrene derivatives **E** in our experiments (Table 1). Indeed, copper hydrides are known to undergo 1,2-addition across alkynes to generate copper vinyl complexes, which by protonolysis give alkenes.^15^ Consistent with this hypothesis, a catalytic experiment using deuterium labelled phenyl acetylene under our optimized conditions, but in CD_2_Cl_2_ instead of C_6_D_6_ (Table 1, entry 6), afforded **B_3_** and **Styrene-D** (75% D-incorporation) (Scheme 4).

**Scheme 4:**
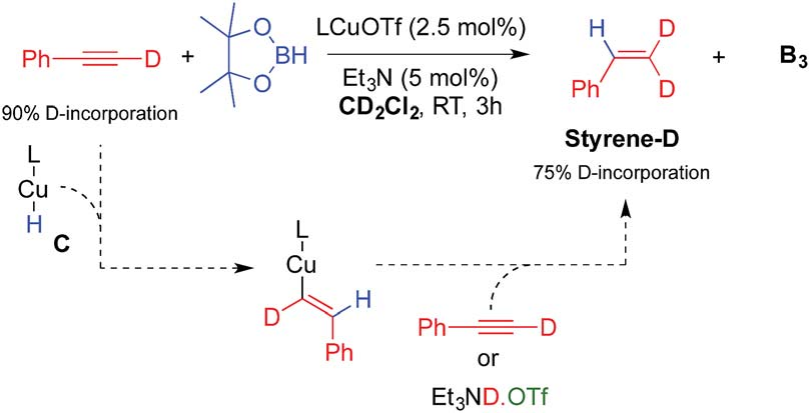
Evidence for the formation of the copper hydride **C**.

## Conclusions

In summary, we have disclosed the first example of a highly selective dehydrogenative borylation of terminal alkynes with pinacolborane, using an inexpensive metal center supported by readily accessible ligands.^16^ Preliminary mechanistic studies suggest the pivotal role of a σ,π-bis(copper) acetylide **A** and a copper hydride **C**.

## References

[cit1] Hartwig J. F., Lennox A. J., Lloyd-Jones G. C. (2012). Acc. Chem. Res..

[cit2] Chen H., Schlecht S., Semple T. C., Hartwig J. F. (2000). Science.

[cit3] Cho J. Y., Tse M. K., Holmes D., Maleczka Jr. R. E., Smith III M. R. (2002). Science.

[cit4] Hartwig J. F., Mkhalid I. A., Barnard J. H., Marder T. B., Murphy J. M., Hartwig J. F. (2011). Chem. Soc. Rev..

[cit5] Lee C. I., Zhou J., Ozerov O. V., Lee C. I., Hirscher N. A., Zhou J., Bhuvanesh N., Ozerov O. V., Lee C. I., Shih W. C., Zhou J., Reibenspies J. H., Ozerov O. V., Lee C. I., DeMott J. C., Pell C. J., Christopher A., Zhou J., Bhuvanesh N., Ozerov O. V., Pell C. J., Ozerov O. V. (2013). J. Am. Chem. Soc..

[cit6] Tsuchimoto T., Utsugi H., Sugiura T., Horio S. (2015). Adv. Synth. Catal..

[cit7] Barbeyron R., Benedetti E., Cossy J., Vasseur J. J., Arseniyadis S., Smietana M. (2014). Tetrahedron.

[cit8] Jin L., Tolentino D. R., Melaimi M., Bertrand G., Jin L., Romero E. A., Melaimi M., Bertrand G. (2015). Sci. Adv..

[cit9] For reviews on CAACs, see: Melaimi M. Soleilhavoup M. Bertrand G., Angew. Chem., Int. Ed., 2010, 49 , 8810 Martin D. Melaimi M. Soleilhavoup M. Bertrand G., Organometallics, 2011, 30 , 5304 Soleilhavoup M. Bertrand G., Acc. Chem. Res., 2015, 48 , 256 .

[cit10] For the synthesis of CAACs, see: Lavallo V. Canac Y. Präsang C. Donnadieu B. Bertrand G., Angew. Chem., Int. Ed., 2005, 44 , 5705 Jazzar R. Dewhurst R. D. Bourg J. B. Donnadieu B. Canac Y. Bertrand G., Angew. Chem., Int. Ed., 2007, 46 , 2899 Jazzar R. Bourg J. B. Dewhurst R. D. Donnadieu B. Bertrand G., J. Org. Chem., 2007, 72 , 3492 .

[cit11] For other mechanistic studies, involving the transient formation of σ,π-bis(copper) acetylides, see: Rodionov V. O. Fokin V. V. Finn M. G., Angew. Chem., Int. Ed., 2005, 44 , 2210 Himo F. Lovell T. Hilgraf R. Rostovtsev V. V. Noodleman L. Sharpless K. B. Fokin V. V., J. Am. Chem. Soc., 2005, 127 , 210 Ahlquist M. Fokin V. V., Organometallics, 2007, 26 , 4389 Straub B. F., Chem. Commun., 2007 , 3868 Nolte C. Mayer P. Straub B. F., Angew. Chem., Int. Ed., 2007, 46 , 2101 Makarem A. Berg R. Rominger F. Straub B. F., Angew. Chem., Int. Ed., 2015, 54 , 7431 Worrell B. T. Malik J. A. Fokin V. V., Science, 2013, 340 , 457 .

[cit12] Arduengo A. J., Krafczyk R., Schmutzler R. (1999). Tetrahedron.

[cit13] For reviews, see: Egbert J. D. Cazin C. S. J. Nolan S. P., Catal. Sci. Technol., 2013, 3 , 912 Deutsch C. Krause N. Lipshutz B. H., Chem. Rev., 2008, 108 , 2916 Lipshutz B. H., Synlett, 2009 , 509 .

[cit14] For an example of diligated copper hydride, see: Lipshutz B. H. Frieman B. A., Angew. Chem., Int. Ed., 2005, 44 , 6345 . 10.1002/anie.200500800 16124024

[cit15] Frey G. D., Donnadieu B., Soleilhavoup M., Bertrand G., Cox N., Dang H., Whittaker A. M., Lalic G., Uehling M. R., Rucker R. P., Lalic G., Suess A. M., Uehling M. R., Kaminsky W., Lalic G., Mankad N. P., Laitar D. S., Sadighi J. P., Semba K., Fujihara T., Xu T. H., Terao J., Tsuji Y., Jordan A. J., Wyss C. M., Bacsa J., Sadighi J. P., Suess A. M., Lalic G., Collins L. R., Riddlestone I. M., Mahon M. F., Whittlesey M. K., Wyss C. M., Tate B. K., Bacsa J., Gray T. G., Sadighi J. P. (2011). Chem.–Asian J..

[cit16] CAAC and NHC precursors are commercially available

