# Supplementary material for: Copper-catalyzed dehydrogenative borylation of terminal alkynes with pinacolborane
Source: Chem Sci. 2016 Aug 9;8(1):165–8. doi: 10.1039/c6sc02668k (PMC5308285; doi:10.1039/c6sc02668k)
Supplement: Supplementary file 1 [file SC-008-C6SC02668K-s001.pdf]

Electronic Supplementary Material (ESI) for Chemical Science.
This journal is © The Royal Society of Chemistry 2016

Copper-Catalyzed Dehydrogenative Borylation of Alkynes Terminal Alkynes with Pinacolborane

Erik A. Romero, Rodolphe Jazzar, Guy Bertrand*

UCSD-CNRS Joint Research Laboratory (UMI 3555), Department of Chemistry and Biochemistry, University of California, San Diego, La Jolla, California 92093-0358, United States

A. General Considerations 2
B. Optimization Tables for the Dehydroborylation Reaction 2
C. Catalytic procedure for the Dehydroborylation Reaction 3
D. Compounds B_1-B_{15} 4
E. Preliminary mechanistic studies 6
F. Spectra 13

A. General Considerations

All reactions were performed under an atmosphere of argon using standard Schlenk or dry box techniques; solvents were dried over Na metal, or CaH_2. Reagents of analytical grade were obtained from commercial suppliers, dried over 4Å molecular sieves and degassed before use. ^{1}H, ^{13}C, and ^{19}F NMR spectra were obtained with a Bruker Advance 300 MHz, and a Varian INOVA 500 MHz spectrometer. Chemical shifts (δ) are reported in parts per million (ppm) relative to TMS, and were referenced to the residual solvent peak. NMR multiplicities are abbreviated as follows: s = singlet, d = doublet, t = triplet, q = quartet, quin = quintet, sex sextet, m = multiplet, br = broad signal. Complexes (Et_2CAAC)CuOTf (**L_1CuOTf**),[i](MenthylCAAC)CuOTf (**L_2CuOTf**),[i] (IPr)CuOTf (**L_3CuOTf**)[ii] were prepared following literature procedures.

B. Optimization Tables for the Dehydroborylation Reaction

Table S1. In a culture tube were added equipped with a magnetic stir bar **cat.** (x mol%) and 4-tolylacetylene (0.087 ml, 0.69 mmol) were combined in a culture tube. y µL (determined according to the named concentration **M**) of **Solvent** was added followed immediately by appropriate amount of base additive (z mol%). After 1 minute stirring, HBPin (0.100 ml, 0.69 mmol) was added in a single portion and the resulting solution was allowed to stir for 2 hours at room temperature. NMR yields based on 1 equivalent addition of 1,4-dioxane after the 2 hour stirring period.

Entry	Cat. (mol%)	Base (mol%)	Solvent	Conc. (M)	1a (%)^{b}	B_1 (%)^{b}	D_1 (%)^{b}	E_1 (%)^{b}
1	-	-	C_6D_6	1.4	100	0	0	0
2	L_1CuOTf (1)	-	C_6D_6	1.4	86	0	6	6
3	L_1CuOTf (1)	Et_3N (1)	C_6D_6	1.4	14	48	11	7
4	CuOTf	Et_3N (1)	C_6D_6	1.4	100	0	0	0
5	L_1CuOTf (1)	Et_3N (2)	C_6D_6	1.4	1	70	14	12
6	L_1CuOTf (1)	Et_3N (2)	CD_2Cl_2	1.4	12	42	6	26
7	L_1CuOTf (1)	Et_3N (2)	THF-d_8	1.4	5	64	12	17
8	L_1CuOTf (1)	Et_3N (2)	CD_3CN	1.4	18	38	0	6
9	L_1CuOTf (1)	$^{i}PrNH_2$ (2)	C_6D_6	1.4	67	5	12	10
10	L_1CuOTf (1)	$^{i}Pr_2NH$ (2)	C_6D_6	1.4	47	11	13	7
11	L_1CuOTf (1)	$^{i}Pr_2NEt$ (2)	C_6D_6	1.4	10	28	45	3
12	L_1CuOTf (1)	$BnNEt_2$ (2)	C_6D_6	1.4	14	53	8	7
13	L_1CuOTf (1)	DABCO (2)	C_6D_6	1.4	18	60	1	7
14	L_1CuOTf (0.25)	Et_3N (0.5)	C_6D_6	1.4	37	36	15	6
15	L_1CuOTf (0.5)	Et_3N (1)	C_6D_6	1.4	20	54	15	9
16	L_1CuOTf (2.5)	Et_3N (5)	C_6D_6	1.4	4	83	4	7
**17**	**L_1CuOTf (2.5)**	**Et_3N (5)**	**C_6D_6**	**0.1**	**1**	**98**	**0**	**1**
18	L_2CuOTf (2.5)	Et_3N (5)	C_6D_6	0.1	0	96	0	4
19	L_3CuOTf (2.5)	Et_3N (5)	C_6D_6	0.1	0	92	0	8

C. Catalytic procedure for the Dehydroborylation Reaction

A General Protocol.

In a schlenk tube under an argon atmosphere were added C_6H_6 (17 mL), the alkyne (1.82 mmol), pinacol borane (0.264 mL, 1.82 mmol), and triethylamine (0.012 mL, 0.091 mmol). Then, Et_2CAACCuOTf (0.024 g, 0.045 mmol%) was added to the mixture and the reaction was left stirring at room temperature until the reaction had completed. After completion, all the volatiles were evaporated under vacuum and 5 mL of pentane was added. The mixture was passed, under argon, through a short column packed with dry neutral alumina (3 cm diameter x 5 cm high) using 30 mL of pentane as eluent. Evaporation of the volatiles under vacuum afforded the corresponding product.

B. Gram Scale Reaction.

In a schlenk tube under an argon atmosphere were added C_6H_6 ([illegible]2 mL, 18.2 mmol), pinacol borane (2.64 mL, 18.2 mmol), and triethylamine (0.12 mL, 0.91 mmol). Then, Et_2CAACCuOTf (0.24 g, 0.45 mmol%) was added to the mixture and the reaction was left stirring at room temperature until the reaction had completed (as confirmed by NMR). After completion, all the volatiles were evaporated under vacuum and 50 mL of pentane was added. The mixture was passed under argon through a short column packed with of dry neutral alumina (3 cm diameter x 5 cm high) using 2 x 25 mL of pentane as eluent. Evaporation of the volatiles under vacuum afforded an analyticaly pure product as a deliquescent solid in 3.952 g (95 %).[iii]

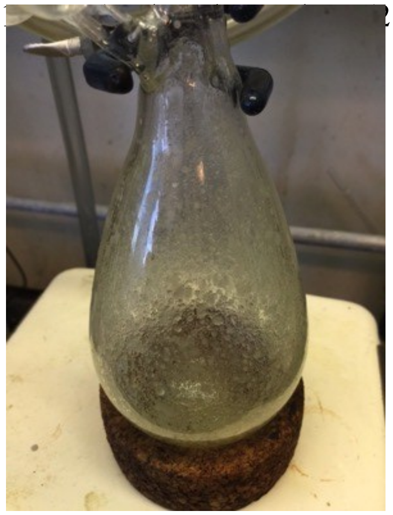

D. Compounds B_1-B_{15}

*NMR spectra of these substrates are in accordance with literature reports (see references accordingly). As noted by others, we were unable to observe the quaternary alkyne carbon (R-***C***CBPin).

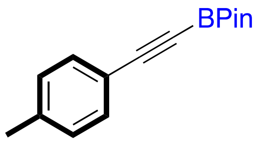

B$_1$[iii,iv]: White solid (m.p. 103-105 ^{o}C) 0.437 g (99% yield). ^{1}H NMR ($CDCl_3$, 500 MHz) δ = 1.27 (s, 12H), 2.29 (s, 3H), 7.07 (d, J = 8.0 Hz, 2H), 7.38 (d, J = 8.0 Hz, 2H) ppm. ^{13}C NMR ($CDCl_3$, 125.7 MHz) δ = 21.5, 24.6, 84.2, 102.0 (br), 118.6, 129.0, 132.5, 139.7 ppm.

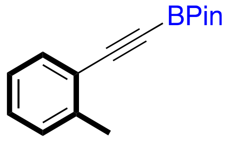

B$_2$[iii]. Deliquescent solid 0.432 g (98% yield). ^{1}H NMR ($CDCl_3$, 500 MHz) δ = 1.03 (s, 12H), 2.35 (s, 3H), 6.75 (t, J = 8.0 Hz, 1H), 6.79 (d, J = 8.0 Hz, 1H), 6.89 (t, J = 8.0 Hz, 1H), 7.45 (d, J = 8.0 Hz, 2H) ppm. ^{13}C NMR ($CDCl_3$, 125.7 MHz) δ = 20.7, 24.7, 84.1, 101.0 (br), 122.6, 125.9, 129.4, 129.7, 133.3, 141.6 ppm.

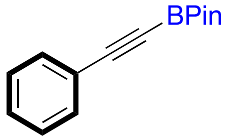

B$_3$[v]: Deliquescent solid 0.411 g (99% yield). ^{1}H NMR ($CDCl_3$, 500 MHz) δ = 1.22 (s, 12H), 7.23 (m, 3H), 7.41 (m, 2H) ppm. ^{13}C NMR ($CDCl_3$, 125.7 MHz) δ = 24.8, 84.6, 101.9 (br), 121.9, 128.4, 129.5, 132.7 ppm.

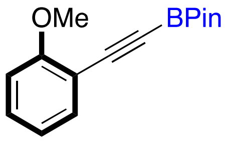

B$_4$: Deliquescent solid 0.413 g (88% yield). ^{1}H NMR (C_6H_6, 500 MHz) δ = 1.00 (s, 12H), 3.19 (s, 3H), 6.31 (d, J = 8.0 Hz, 1H), 6.55 (t, J = 8.0 Hz, 1H), 6.91 (t, J = 8.0 Hz, 1H), 7.44 (d, J = 8.0 Hz, 1H) ppm. ^{13}C NMR (C_6H_6, 125.7 MHz) δ = 24.7, 55.1, 83.9, 98.5 (br), 110.9, 112.3, 120.4, 130.7, 134.7, 160.6 ppm. HRMS analysis was unsuccessful.

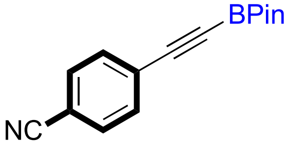

B$_5$: Deliquescent solid 0.392 g (85% yield). ^{1}H NMR (C_6D_6, 300 MHz) δ = 1.23 (s, 12H), 7.51 (s, 4H) ppm. ^{13}C NMR ($CDCl_3$, 125.7 MHz) δ = 24.7, 84.5, 99.5, 113.1, 118.2, 126.4, 131.9, 132.6 ppm.

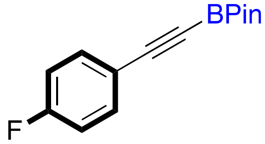

B$_6$[iii,v]: Deliquescent solid 0.367 g (85% yield). ^{1}H NMR ($CDCl_3$, 500 MHz) δ = 1.31 (s, 12H), 7.0 (t, J = 8.7 Hz, 2H), 7.51 (m, 2H) ppm. ^{13}C NMR ($CDCl_3$, 125.7 MHz) δ = 24.7, 84.2, 100.7 (br), 115.5 (d, J = 22.1 Hz), 117.9 (d = 3.5 Hz), 134.7 (J = 8.5 Hz), 163.3 (J = 250.4 Hz) ppm.

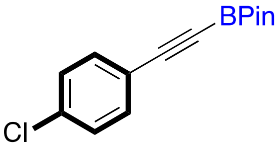

B$_7$[iii,iv]: Deliquescent solid 0.392 g (82% yield). ^{1}H NMR (C_6D_6, 500 MHz) δ = 1.02 (s, 12H), 6.75 (d, J = 8.5 Hz, 2H), 7.03 (d, J = 8.5 Hz, 2H) ppm. ^{13}C NMR (C_6D_6, 125.7 MHz) δ = 24.7, 84.2, 100.6 (br), 121.0, 129.0, 133.9, 135.5 ppm.

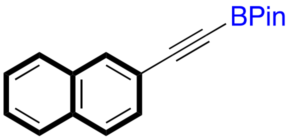

B$_8$[iii]: Deliquescent solid 0.407 g (81% yield). ^{1}H NMR (C_6D_6, 500 MHz) δ = 1.06 (s, 12H), 7.12 (m, 2H), 7.32 (m, 1H), 7.34 (m, 1H), 7.40 (m, 1H), 7.43 (m, 1H), 7.85 (s, 1H) ppm. ^{13}C NMR (C_6D_6, 125.7 MHz) δ = 23.4, 82.8, 101.0 (br), 118.5, 125.4, 125.9, 127.2, 127.4, 131.8, 132.1, 132.3 ppm.

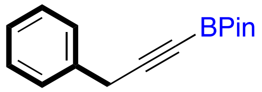

B_9: Deliquescent solid 0.374 g (85% yield). ^1H NMR (C_6D_6, 500 MHz) δ = 1.03 (s, 12H), 3.32 (s, 2H) 7.00-7.20 (m, 5H) ppm. ^{13}C NMR (C_6D_6, 125.7 MHz) δ = 24.7, 25.9, 83.8, 101.6 (br), 126.9, 128.2, 128.7, 135.9 ppm.

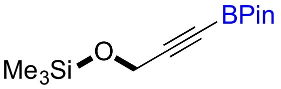

B_{10}[v]: Colorless oil 0.250 g (89% yield). ^1H NMR ($CDCl_3$, 300 MHz) δ = 0.07 (s, 9H), 1.17 (s, 12H), 4.12 (s, 2H) ppm. ^{13}C NMR (C_6D_6, 125.7 MHz) δ = 0.3, 24.7, 51.3, 84.3, 101.2 (br) ppm.

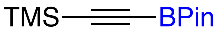

B_{11} [v,vi]: Colorless oil 0.404 g (99% yield). ^1H NMR (C_6D_6, 500 MHz) δ = 0.05 (s, 9H), 0.93 ppm. ^{13}C NMR (C_6D_6, 125.7 MHz) δ = 0.51, 24.6, 84.0, 110.1 (br) ppm.

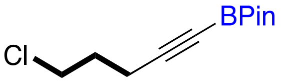

B_{12}[v]: Colorless oil 0.437 g (99% yield). ^1H NMR (C_6D_6, 500 MHz) δ = 1.01, 1.410 (quint, J = 7.0 Hz, 2H), 1.99 (t, J = 7.0 Hz, 2H), 3.09 (t, J = 7.0 Hz, 2H) ppm. ^{13}C NMR (C_6D_6, 125.7 MHz) δ = 16.9, 24.7, 31.0, 43.3, 83.8, 102.3 (br) ppm.

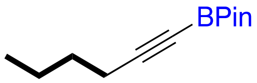

B_{13}[vii]: Colorless oil 0.400 g (99% yield). ^1H NMR (C_6D_6, 500 MHz) δ = 0.67 (t, J = 7.5 Hz, 3H), 1.01 (s, 12H), 1.23 (septet, J = 7.5 Hz, 2H), 1.96 (t, J = 7.5 Hz, 2H) ppm. ^{13}C NMR (C_6D_6, 125.7 MHz) δ = 13.2, 19.0, 21.6, 24.3, 30.0, 83.2, 104.0 (br) ppm.

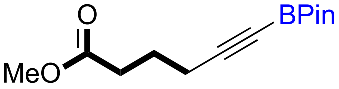

B_{14}: Colorless oil 0.441 g (96% yield). ^1H NMR (C_6D_6, 300 MHz) δ = 1.31 (s, 12H), 1.80 (quint, J = 6.0 Hz, 2H), 2.29 (t, J = 6.0 Hz, 2H), 2.40 (t, J = 6.0 Hz, 2H), 3.61 (s, 3H) ppm. ^{13}C NMR (C_6D_6, 125.7 MHz) δ = 18.9, 23.6, 24.7, 32.5, 51.0, 83.7, 103.2, 172.6 ppm.

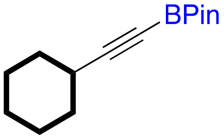

B_{15}[iii,v]: Deliquescent solid 0.405 g (95% yield). ^1H NMR (C_6D_6, 500 MHz) δ =1.26 (m, 16H), 1.00 (m, 13H), 1.19 (m, 1H) 1.38-1.64 (m, 8H), 2.25 (m, 1H) ppm. ^{13}C NMR (C_6D_6, 125.7 MHz) δ = 24.5, 24.6, 25.7, 29.7, 32.1, 83.5, 108.3 (br) ppm.

E. Preliminary Mechanistic Studies

A. Kinetic measurements.

All the kinetic measurements were performed in a J-Young NMR tube at room temperature on a Bruker 300MHz NMR machine using the multi_zgvd command. Conversions were measured by following the growth of the characteristic signals for $\mathbf{B_1}$ or $\mathbf{D_1}$ with respect to the depleting signals for **1a**. In each kinetic run, 0.45 mmol (0.057 mL) of **1a** and 0.45 mmol (0.066 mL) of PinBH were used in combination with 2.5 mol% of the catalyst (either L_1CuOTf: 6.8 mg or L_1CuCCPh: 5.4 mg), the corresponding amount of Et_3N (either 5 mol%: 0.0032 mL or 2.5 mol%: 0.0016 mL) and Et_3NH.OTf (1.4 mg, 2.5 mol%) when needed. At the end of these experiments, the resulting mixture was transferred to a schlenk tube, the volatiles were evaporated under vacuum and 5 mL of pentane was added. The mixture was passed, through a short column packed with dry neutral alumina (3 cm diameter x 5 cm high) under an argon atmosphere using pentane as eluent. Evaporation of the volatiles under vacuum afforded the corresponding product as white solids.

- **Kinetic plots with respect to the formation of B_1**

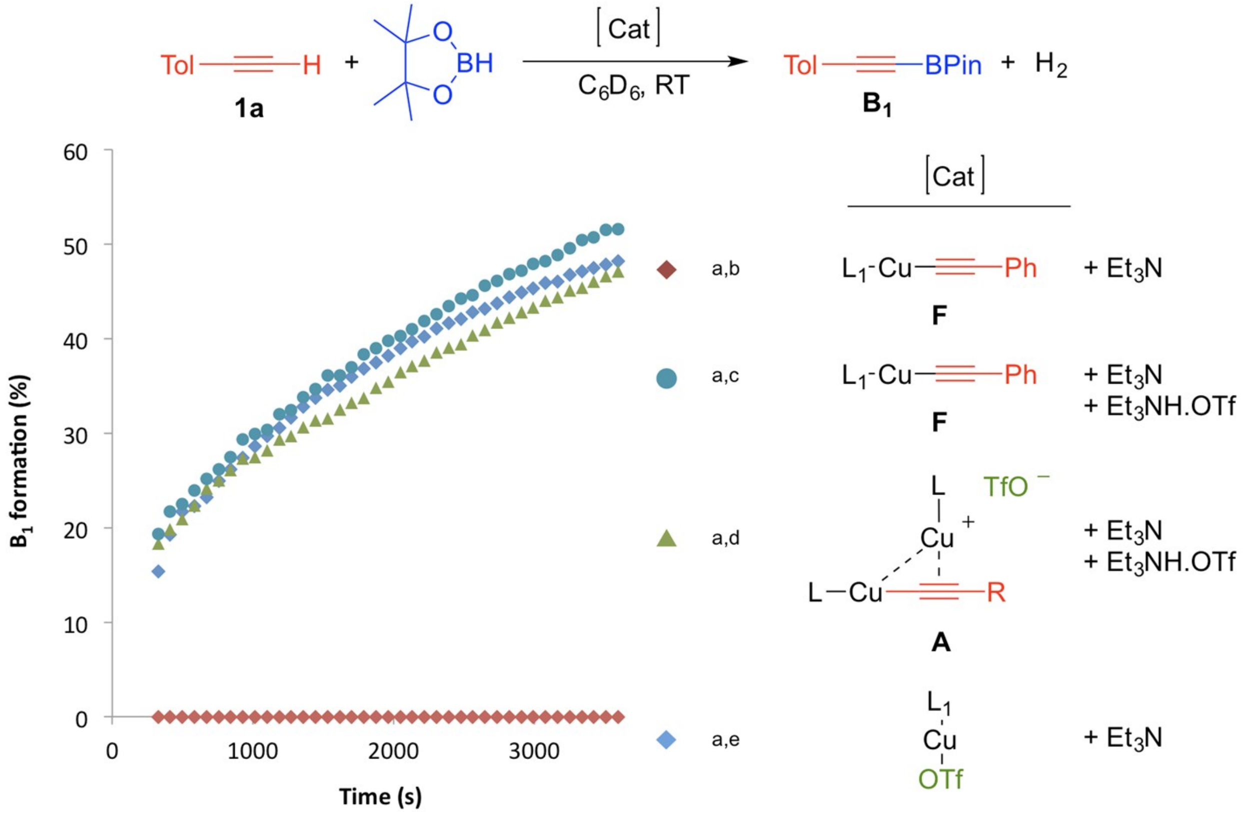

- **Kinetic plot with respect to the formation of D_1**

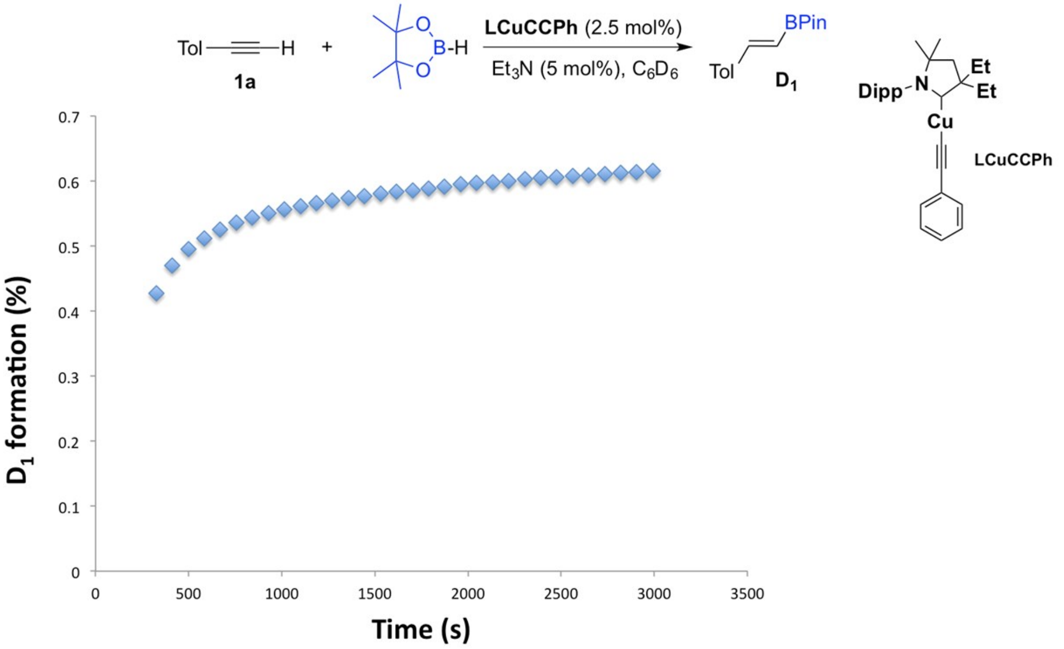

a) Selected spectra from the kinetic run using **Cat**: **$L_1CuCCPh$ + HBPin + Et_3N (2.5 mol%) and $Et_3NH.OTf$ (2.5 mol%)**

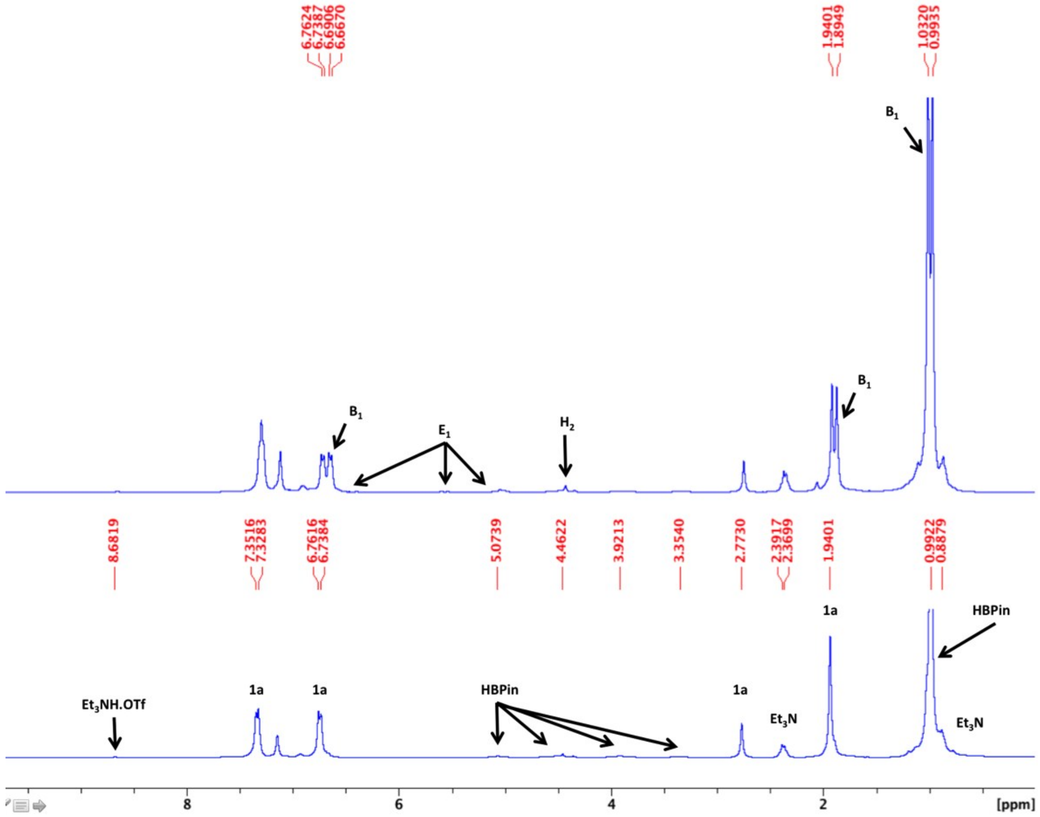

^{1}H NMR spectra after 5 minutes bottom. ^{1}H spectra after 45 min top.

b) Selected spectra from the kinetic run using **Cat**: **$L_1CuCCPh$ + HBPin + Et_3N (5 mol%).**

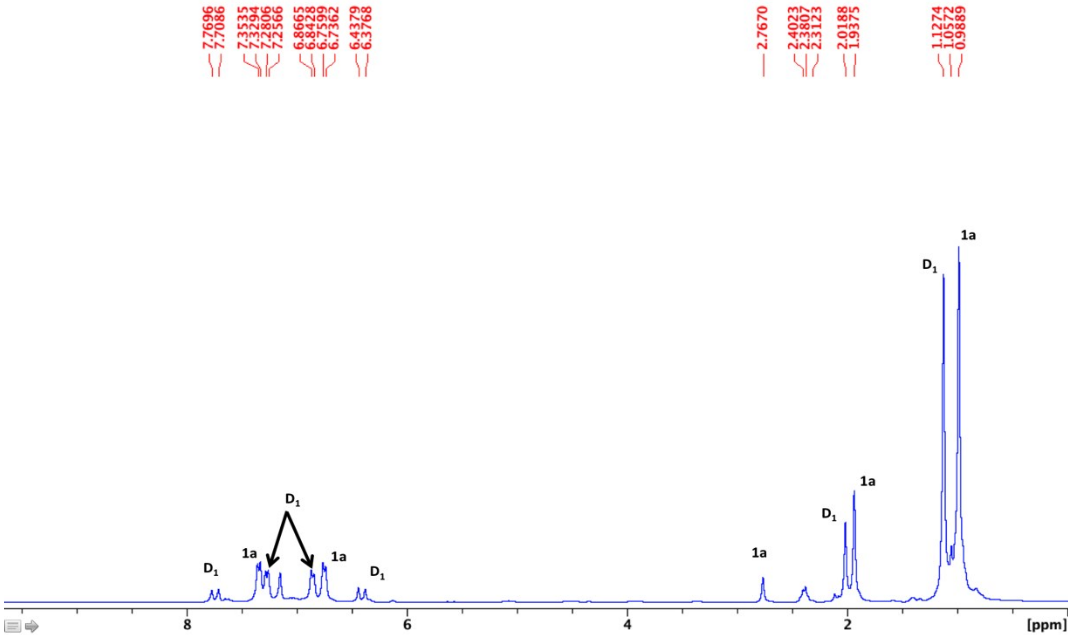

^{1}H NMR spectra after 5 minutes.

NMR of the isolated D_1 isolated at the end of the kinetic run using **Cat**: $L_1CuCCPh$ + HBPin + Et_3N (5 mol%). The reaction was complete within 30 minutes.

Colorless oil : 95% yield. ^{1}H NMR ($CDCl_3$, 300 MHz): δ = 1.26 (s, 12H), 2.28 (s, 3H), 6.11 (d, J = 18.6 Hz, 1H), 7.10 (d, J = 7.9 Hz, 2H), 7.34 (d, J = 7.6 Hz, 2H), 7.38 (d, J = 19.5 Hz, 1H) ppm; ^{13}C NMR ($CDCl_3$, 75 MHz) δ = 21.3, 24.8, 83.2, 115.0 (br), 127.0, 129.3, 134.8, 138.8, 149.5 ppm.

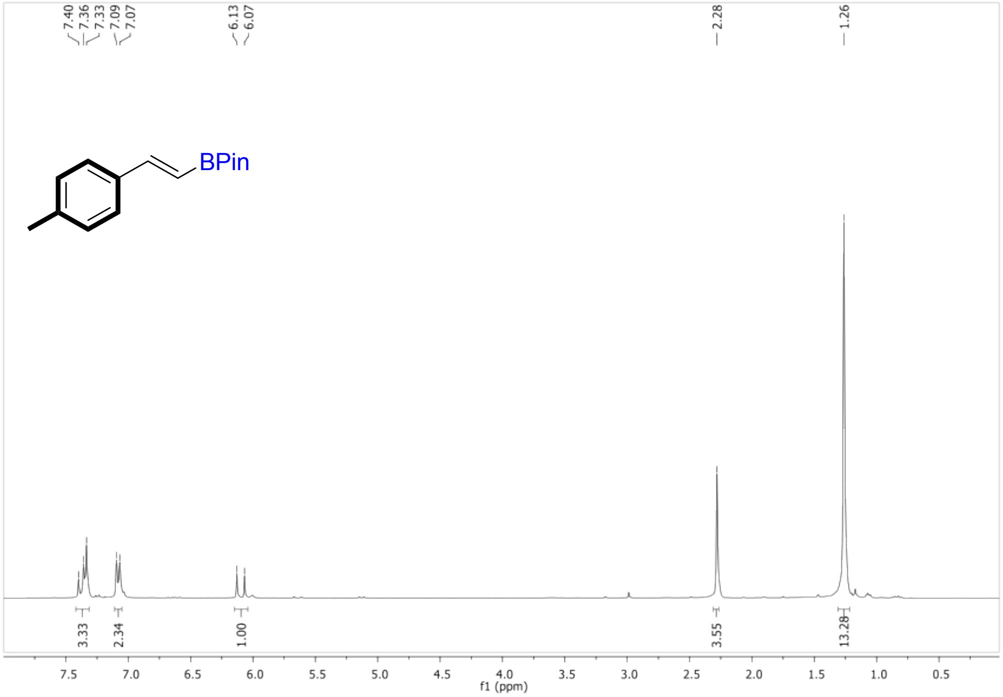

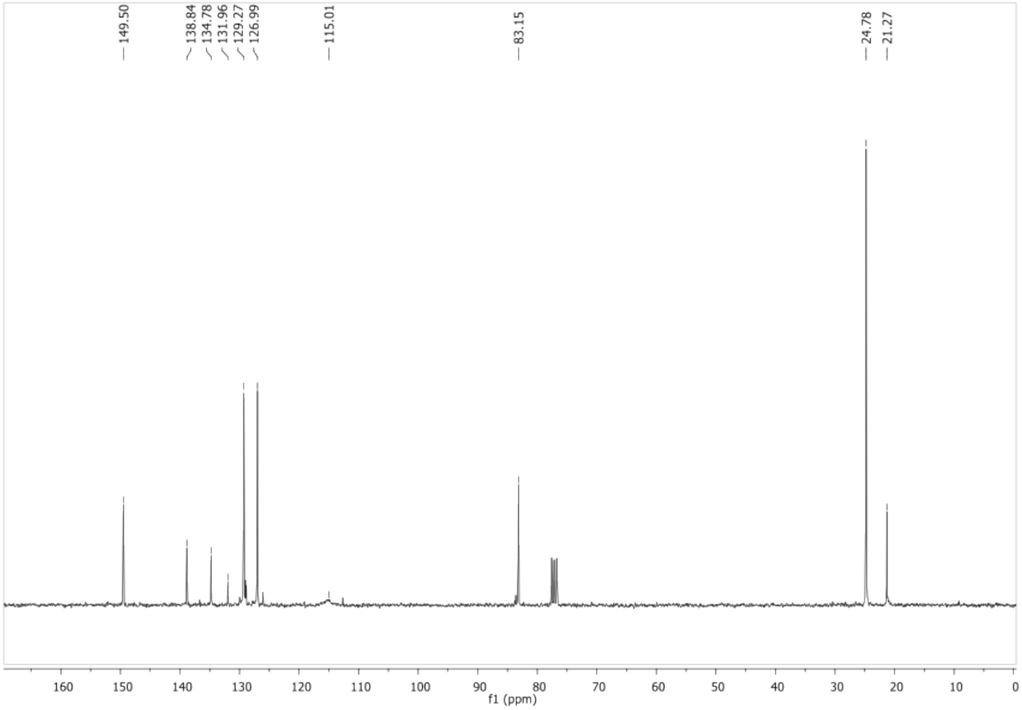

B. Deuterium Labeling for the Dehydrogenative Transformation

In a schlenk tube fitted with a magnetic stir bar were added under an argon atmosphere CD_2Cl_2 (17 mL), D-labeled phenyl acetylene (0.200 ml, 1.82 mmol), pinacol borane (0.264 mL, 1.82 mmol), and triethylamine (0.012 mL, 0.091 mmol). Then, Et_2CAACCuOTf (0.024 g, 0.045 mmol%) was added to the mixture and the reaction was left stirring at room temperature for three hours. After this time NMR-analysis of the content showed the formation of a mixture of $\mathbf{B_1}$ and **styrene-D**.[viii]

^{1}H NMR of Styrene-D

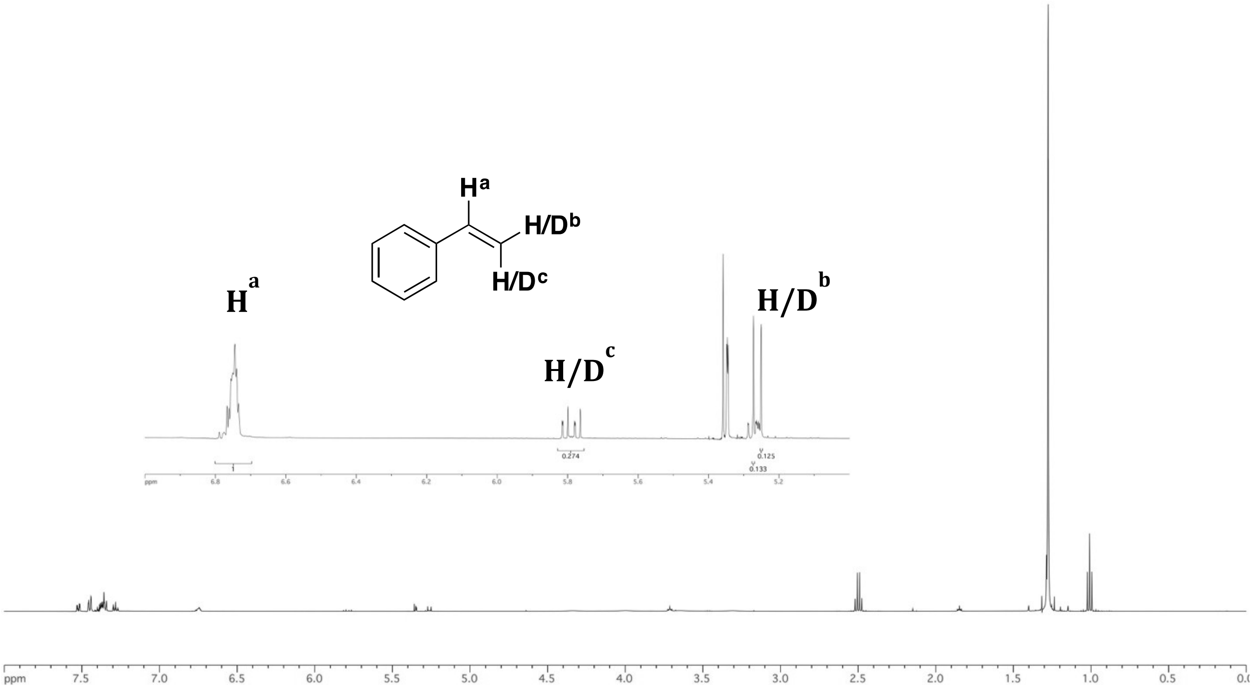

C. Evidence for the formation of A starting from F in the presence of $Et_3NH.OTf$.

A J-Young NMR tube was charged with $CDCl_3$ (1 mL), **F** (20 mg), and $Et_3NH.OTf$ (5.3 mg, 0.5 eq). The NMR taken immediately after mixing the component together show a clean formation of the *bis*-copper **A** and are in agreement with the literature.[i] See NMR spectra bellow.

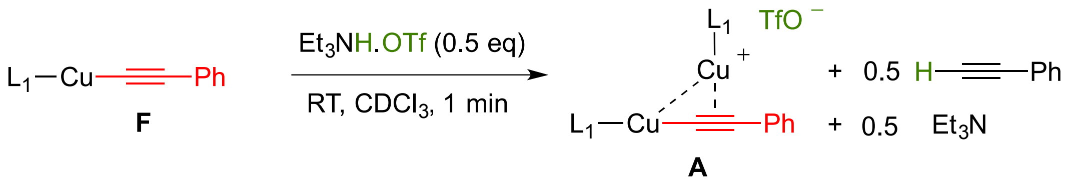

[1]H

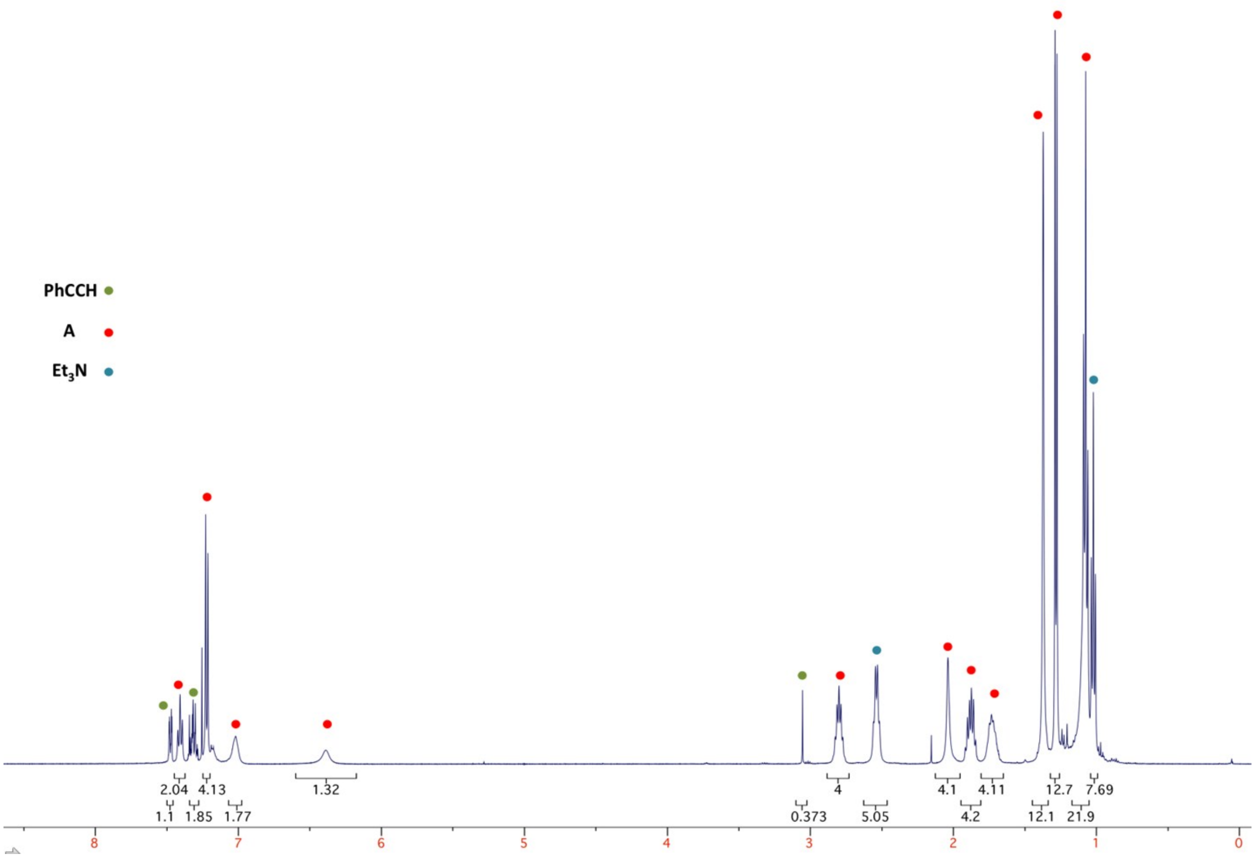

^{13}C

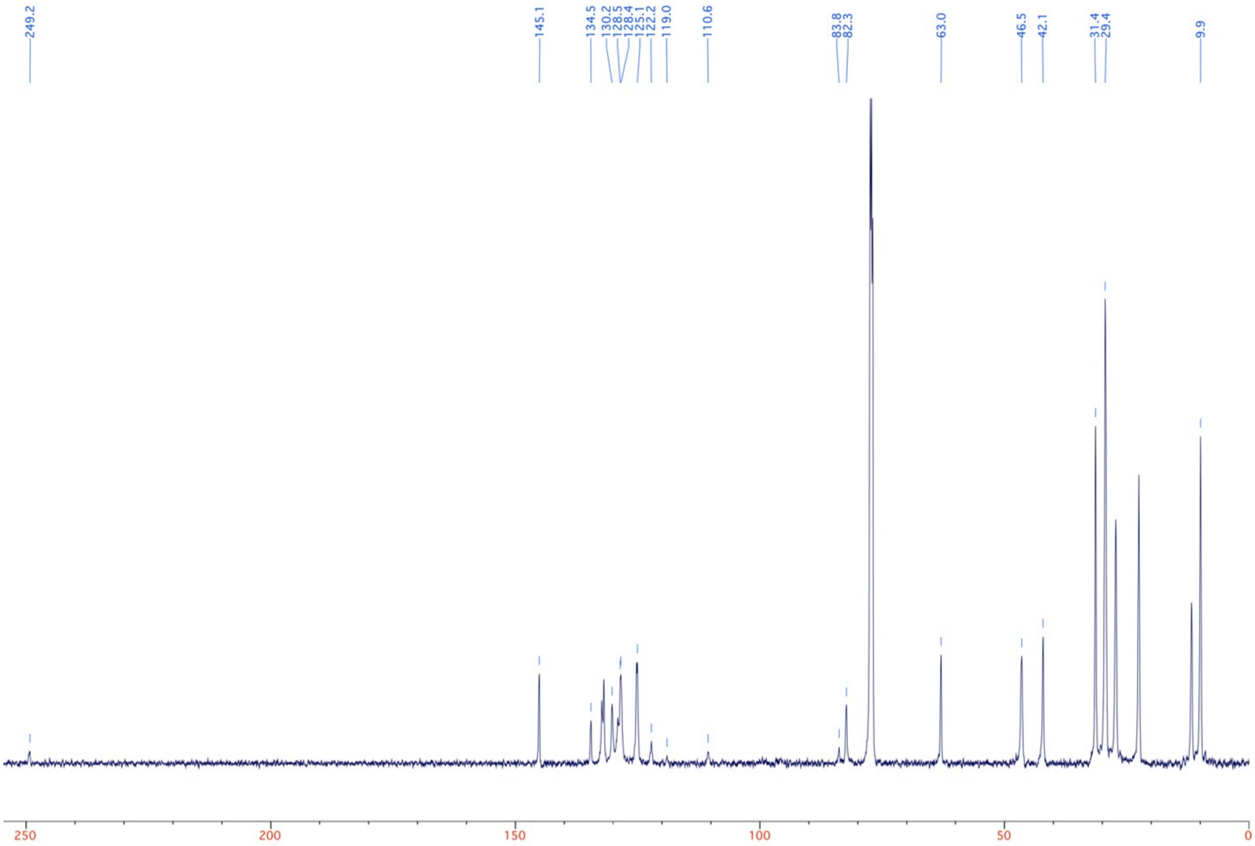
249.2
145.1
134.5
130.2
128.5
128.4
125.1
122.2
119.0
110.6
83.8
82.3
63.0
46.5
42.1
31.4
29.4
9.9
250
200
150
100
50
0

F. Spectra

B_1

^1H

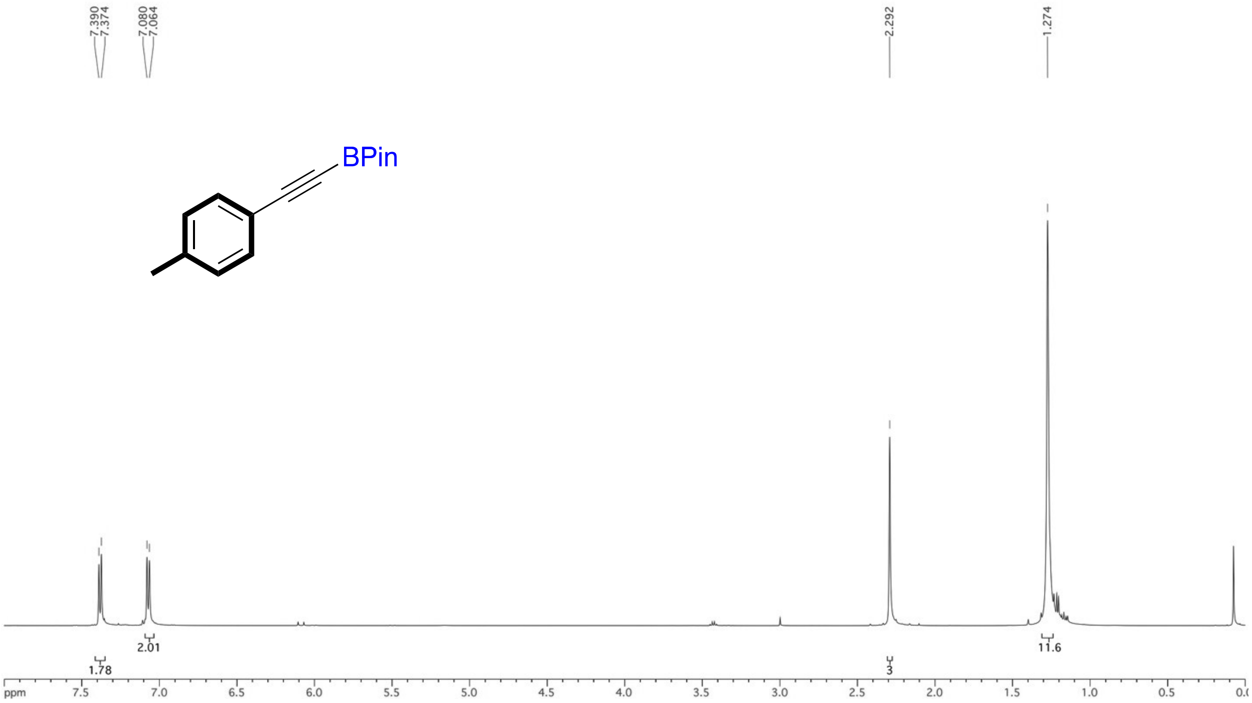

^{13}C

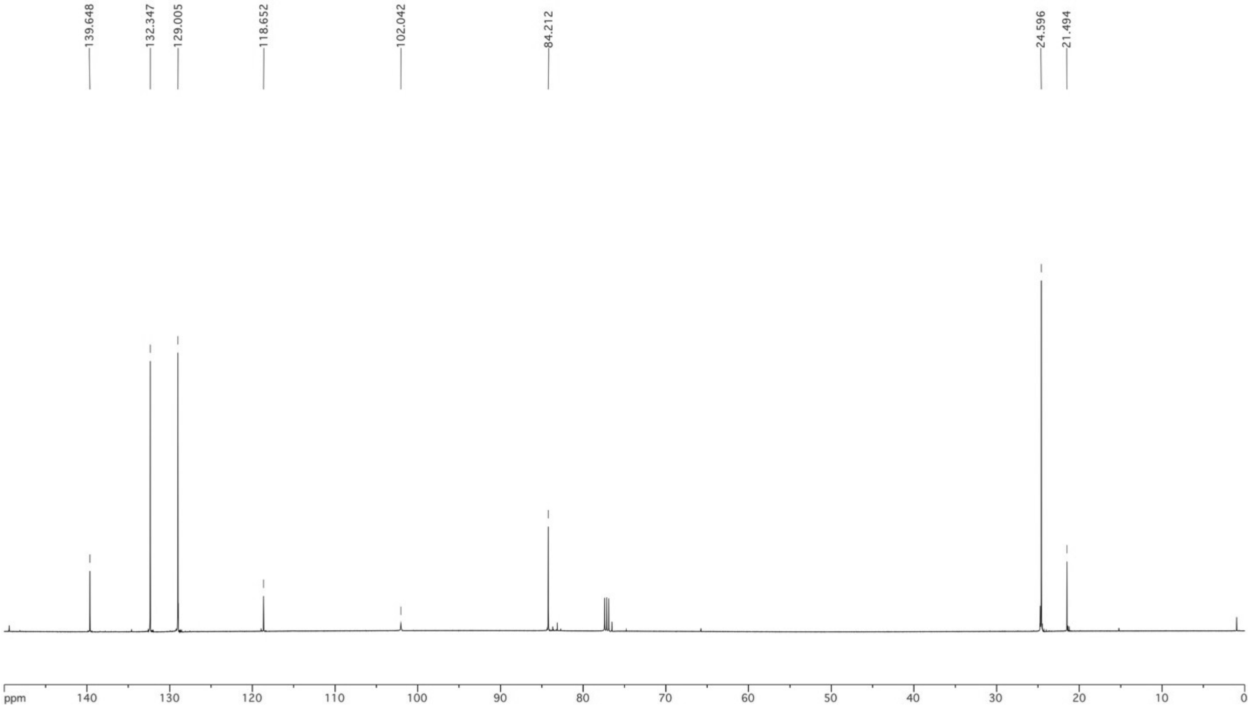

B_2

^{1}H

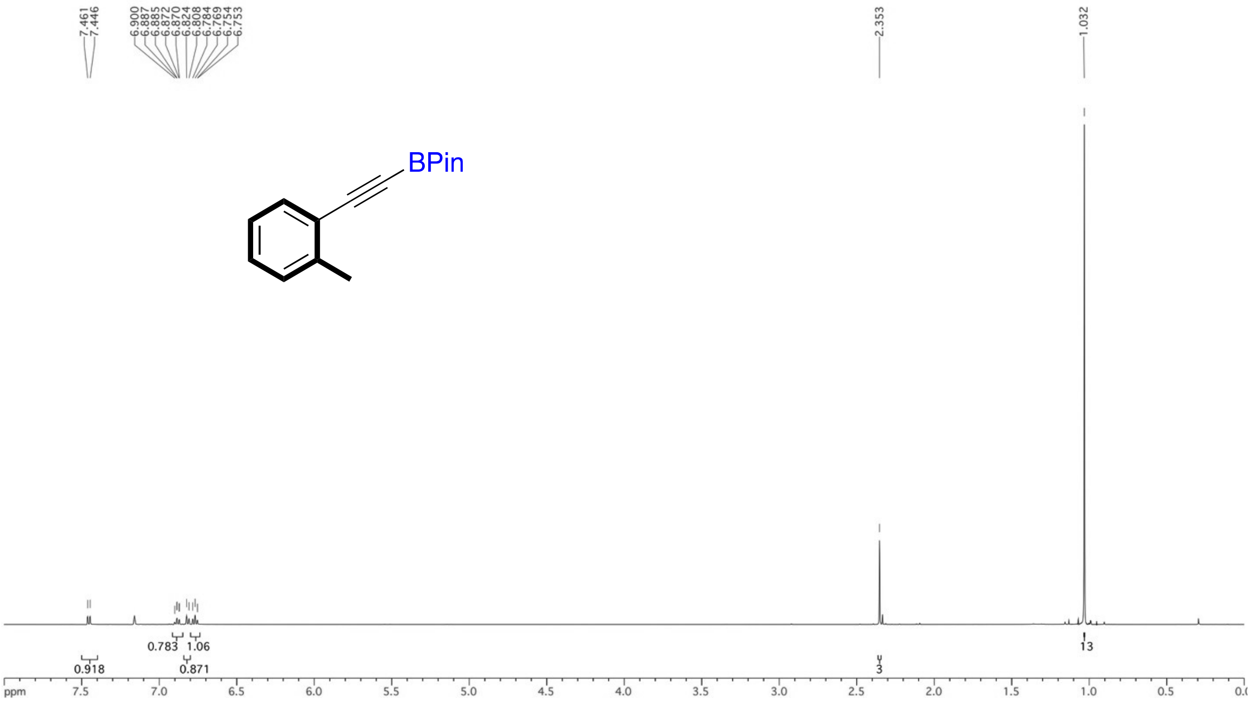
7.461
7.446
6.900
6.887
6.885
6.872
6.870
6.824
6.808
6.784
6.769
6.754
6.753
2.353
1.032
BPin
0.783
1.06
0.918
0.871
3
13
ppm
7.5
7.0
6.5
6.0
5.5
5.0
4.5
4.0
3.5
3.0
2.5
2.0
1.5
1.0
0.5

^{13}C

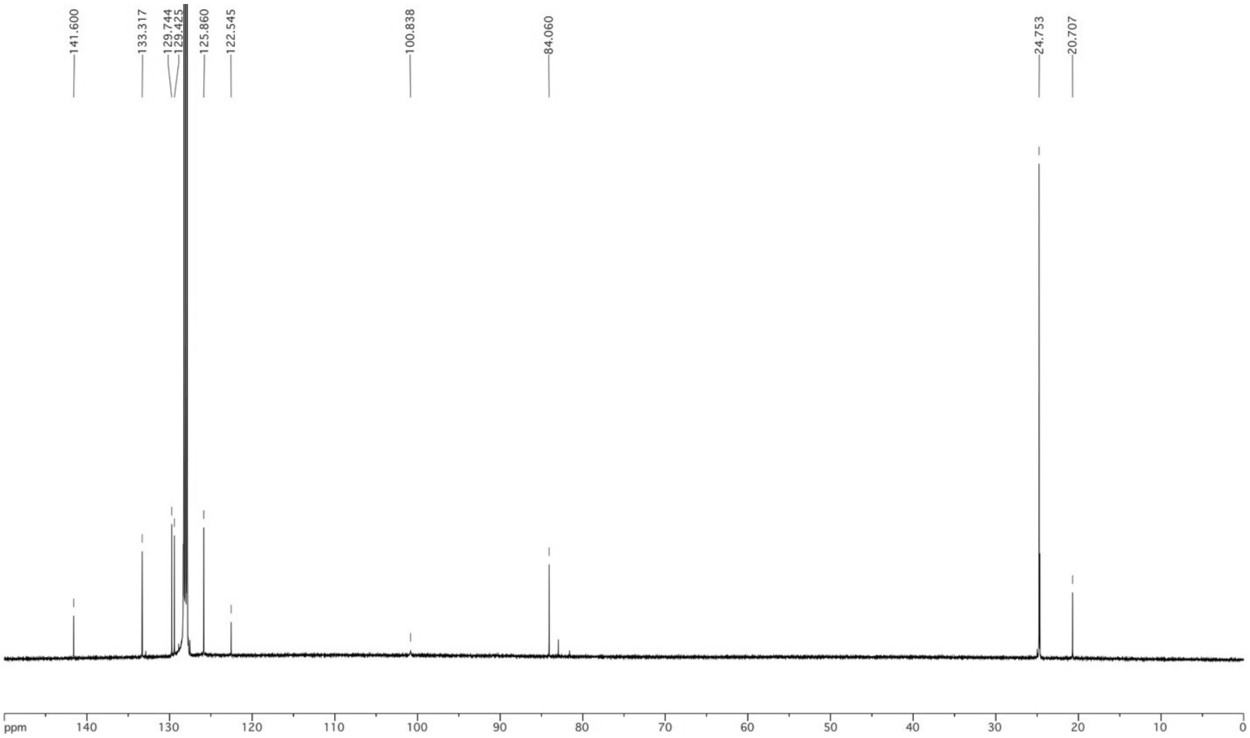
141.600
133.317
129.744
129.425
125.860
122.545
100.838
84.060
24.753
20.707
ppm
140
130
120
110
100
90
80
70
60
50
40
30
20
10
0

B_3

^{1}H

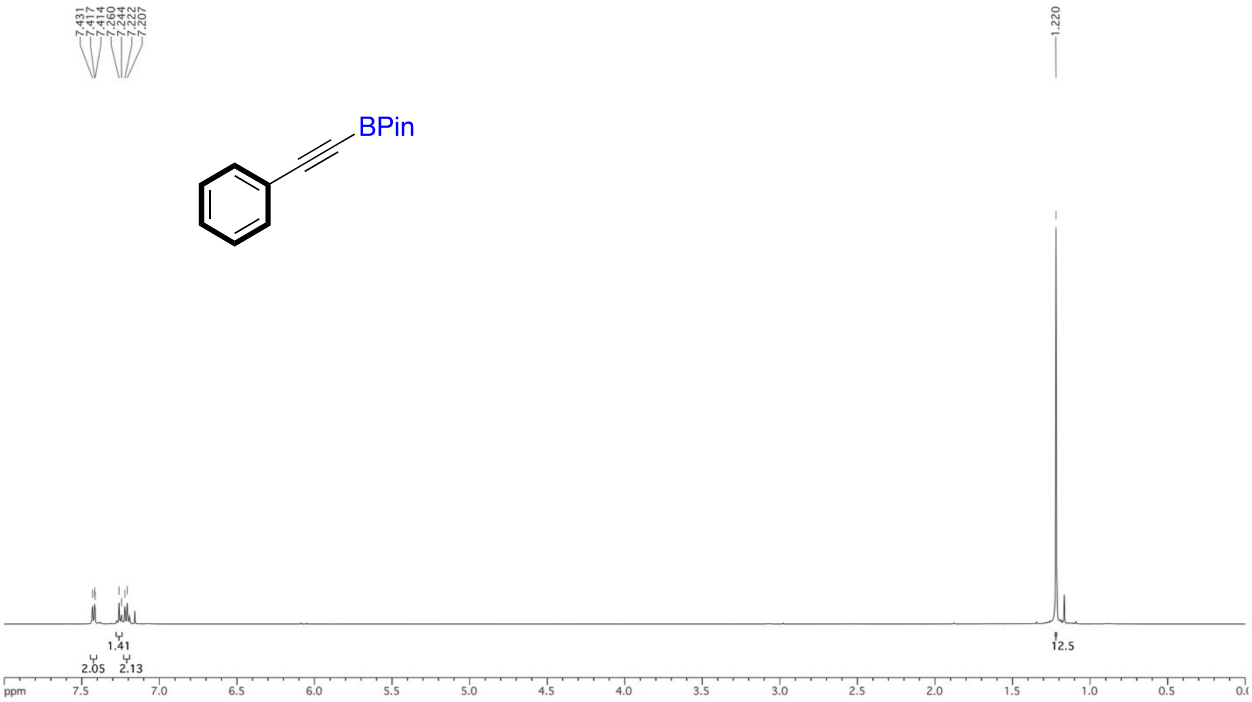
7.431
7.417
7.414
7.260
7.244
7.222
7.207
1.220
BPin
1.41
2.05
2.13
12.5
ppm 7.5 7.0 6.5 6.0 5.5 5.0 4.5 4.0 3.5 3.0 2.5 2.0 1.5 1.0 0.5

^{13}C

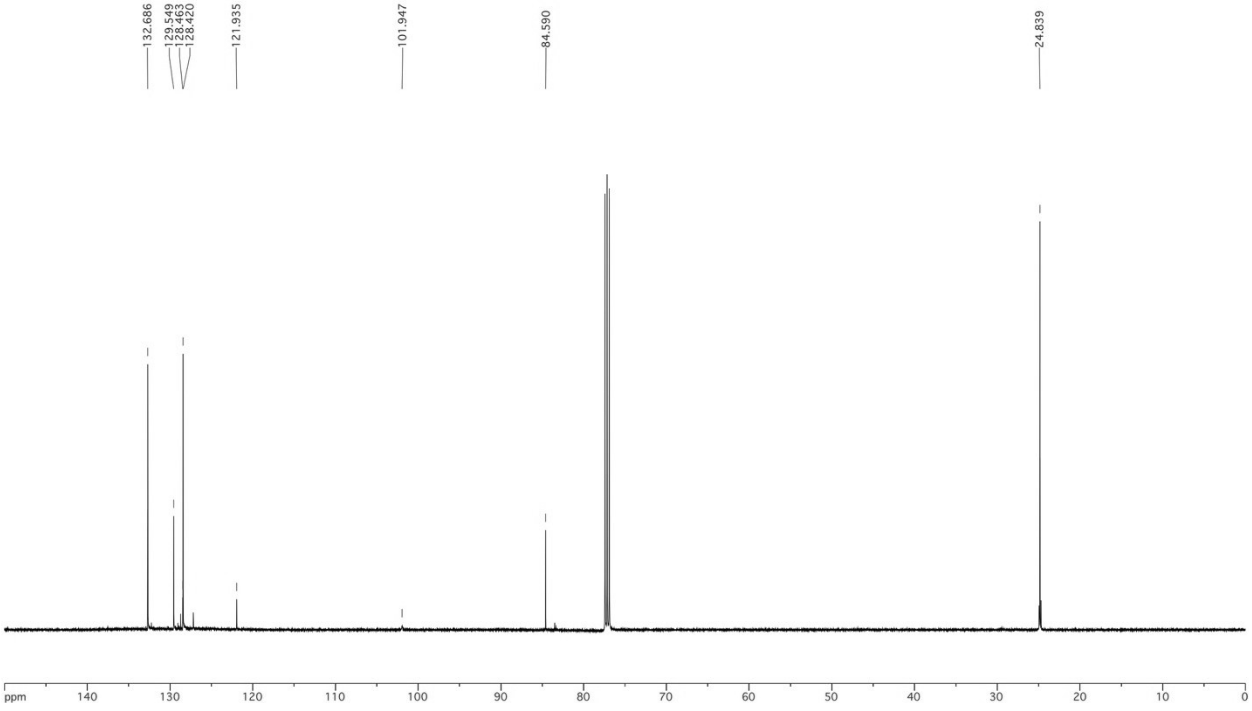
132.686
129.549
128.463
128.420
121.935
101.947
84.590
24.839
ppm 140 130 120 110 100 90 80 70 60 50 40 30 20 10 0

B_4

^{1}H

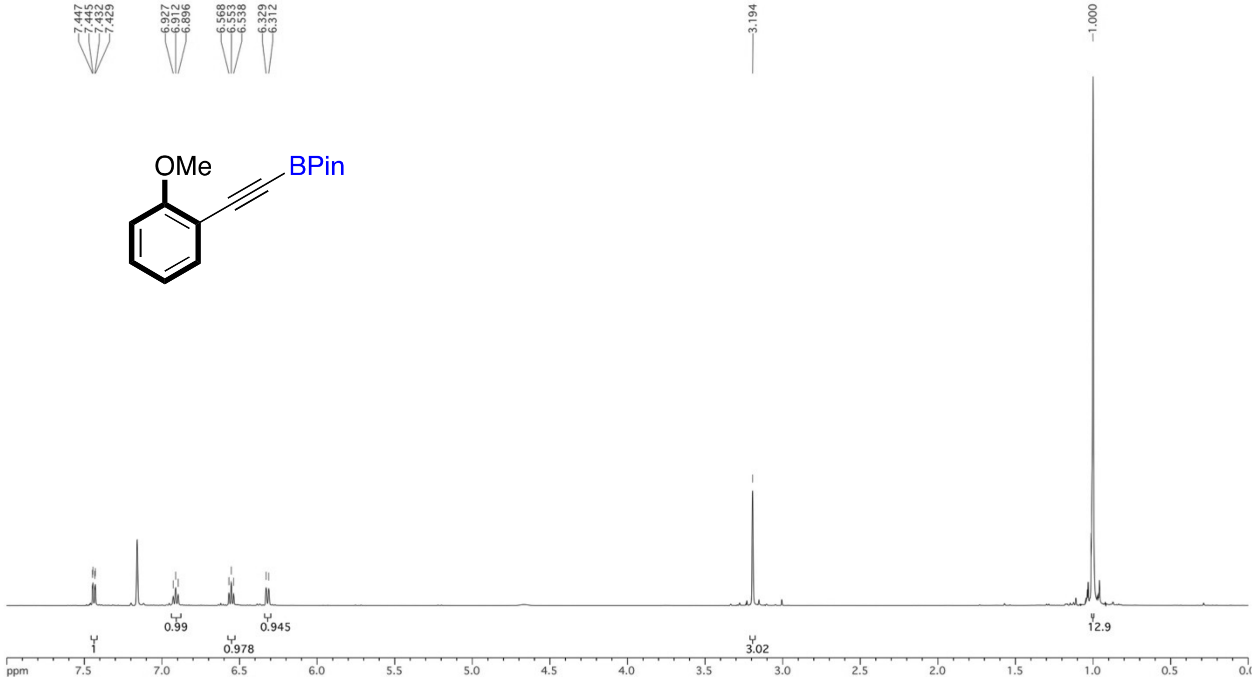

7.447
7.445
7.432
7.429
6.927
6.912
6.896
6.568
6.553
6.538
6.329
6.312
3.194
1.000
OMe
BPin
0.99
0.945
1
0.978
3.02
12.9
ppm
7.5
7.0
6.5
6.0
5.5
5.0
4.5
4.0
3.5
3.0
2.5
2.0
1.5
1.0
0.5

^{13}C

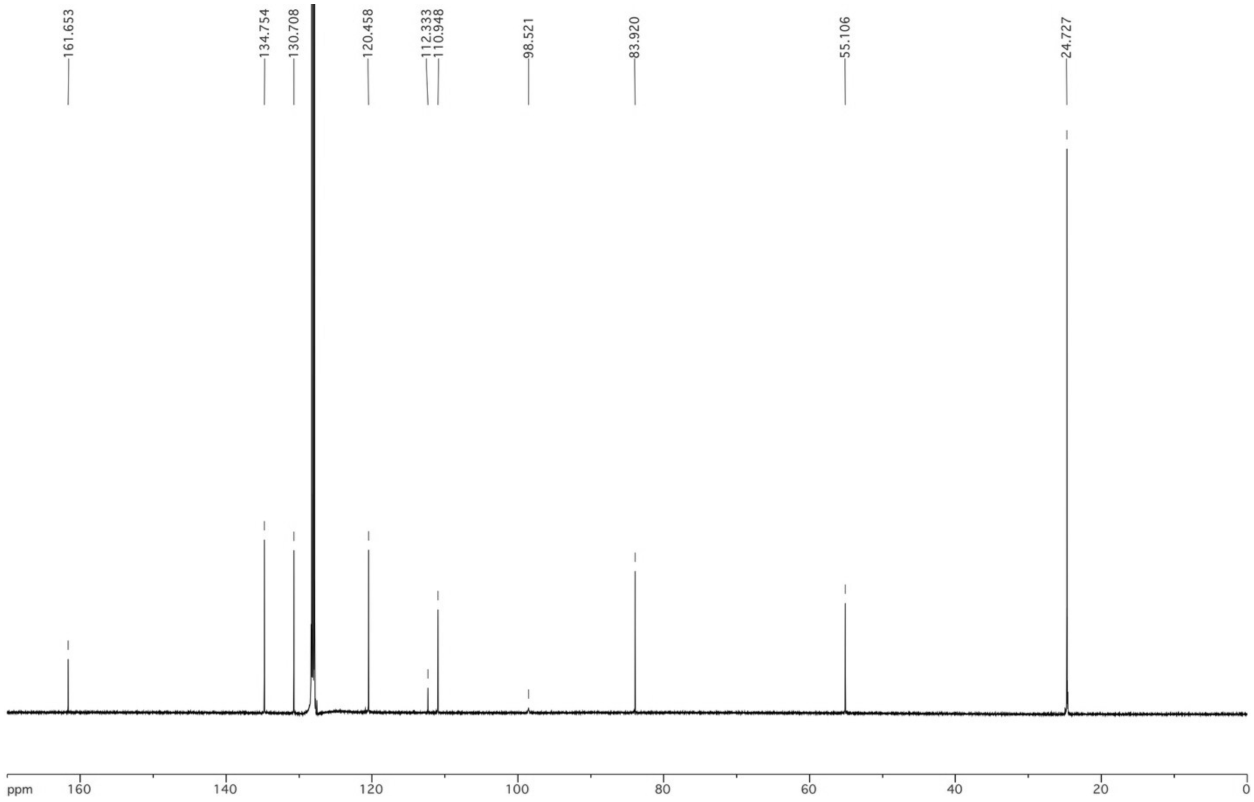

161.653
134.754
130.708
120.458
112.333
110.948
98.521
83.920
55.106
24.727
ppm
160
140
120
100
80
60
40
20
0

B_5

^{1}H

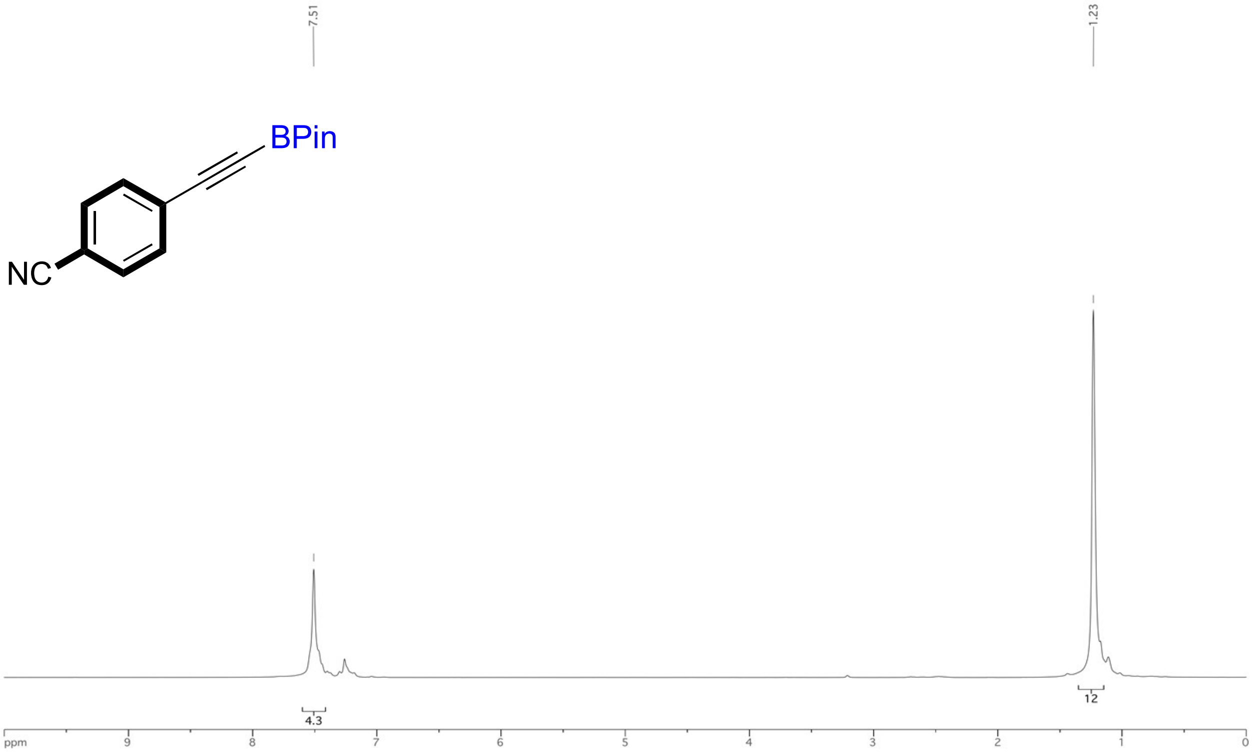
7.51
1.23
BPin
NC
4.3
12
ppm 9 8 7 6 5 4 3 2 1 0

^{13}C

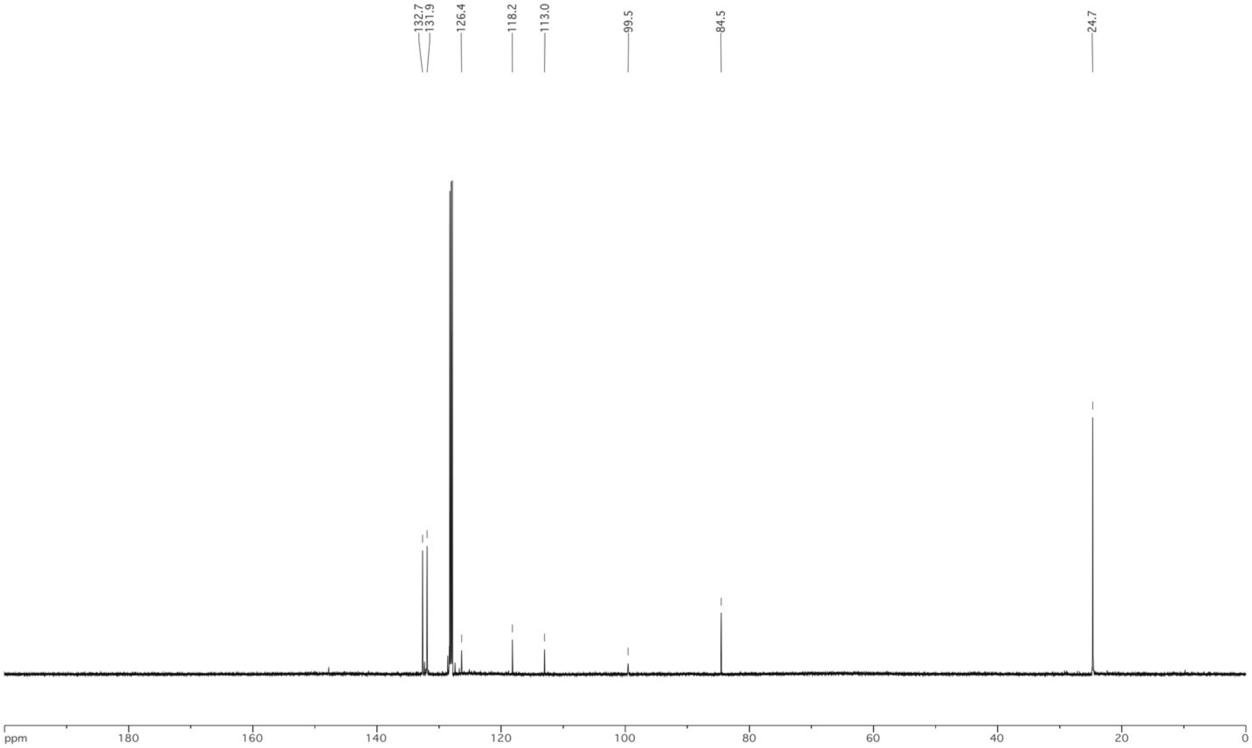
132.7
131.9
126.4
118.2
113.0
99.5
84.5
24.7
ppm 180 160 140 120 100 80 60 40 20 0

B_6

^{1}H

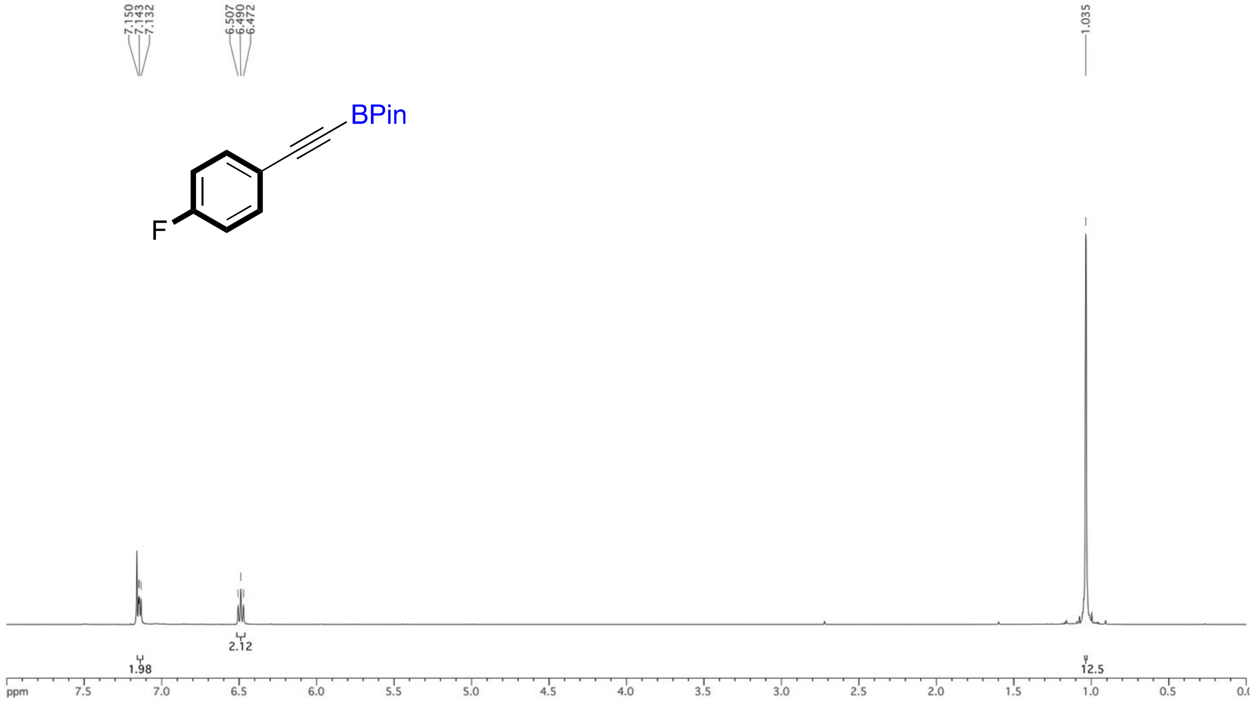
7.150
7.143
7.132
6.507
6.490
6.472
1.035
BPin
F
1.98
2.12
12.5
ppm 7.5 7.0 6.5 6.0 5.5 5.0 4.5 4.0 3.5 3.0 2.5 2.0 1.5 1.0 0.5

^{13}C

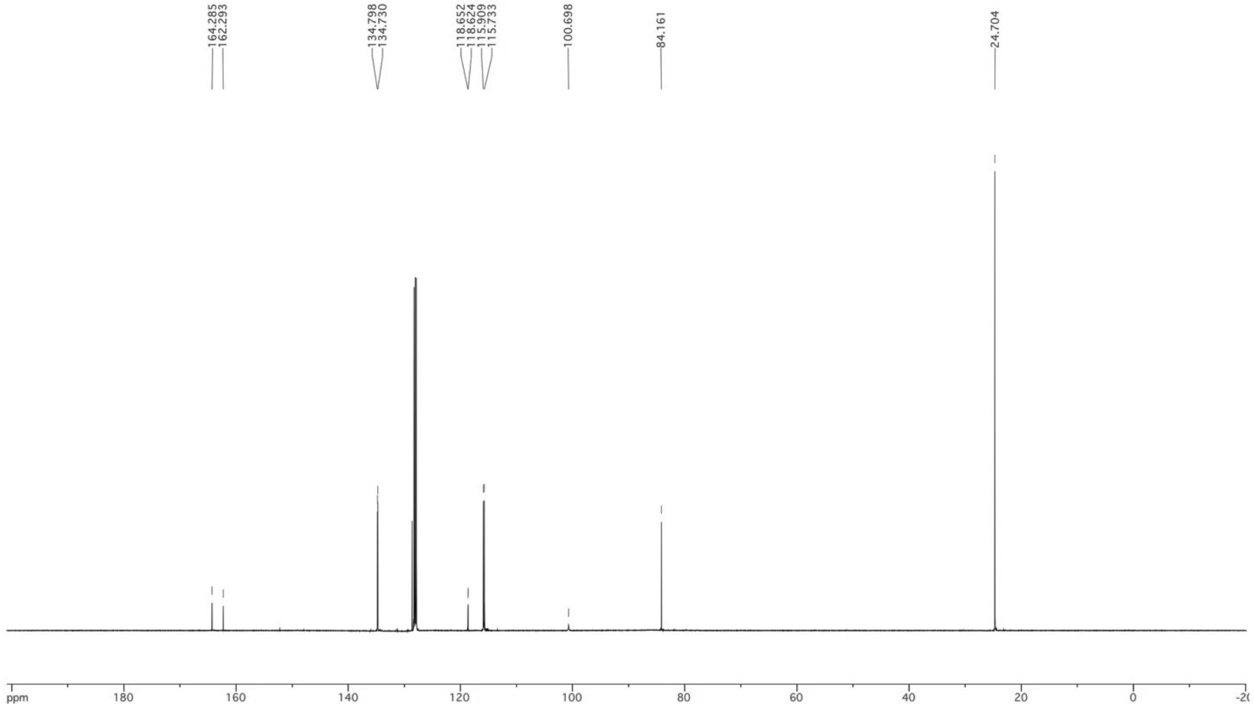
164.285
162.293
134.798
134.730
118.652
118.624
115.909
115.733
100.698
84.161
24.704
ppm 180 160 140 120 100 80 60 40 20 0

B_7

^{1}H

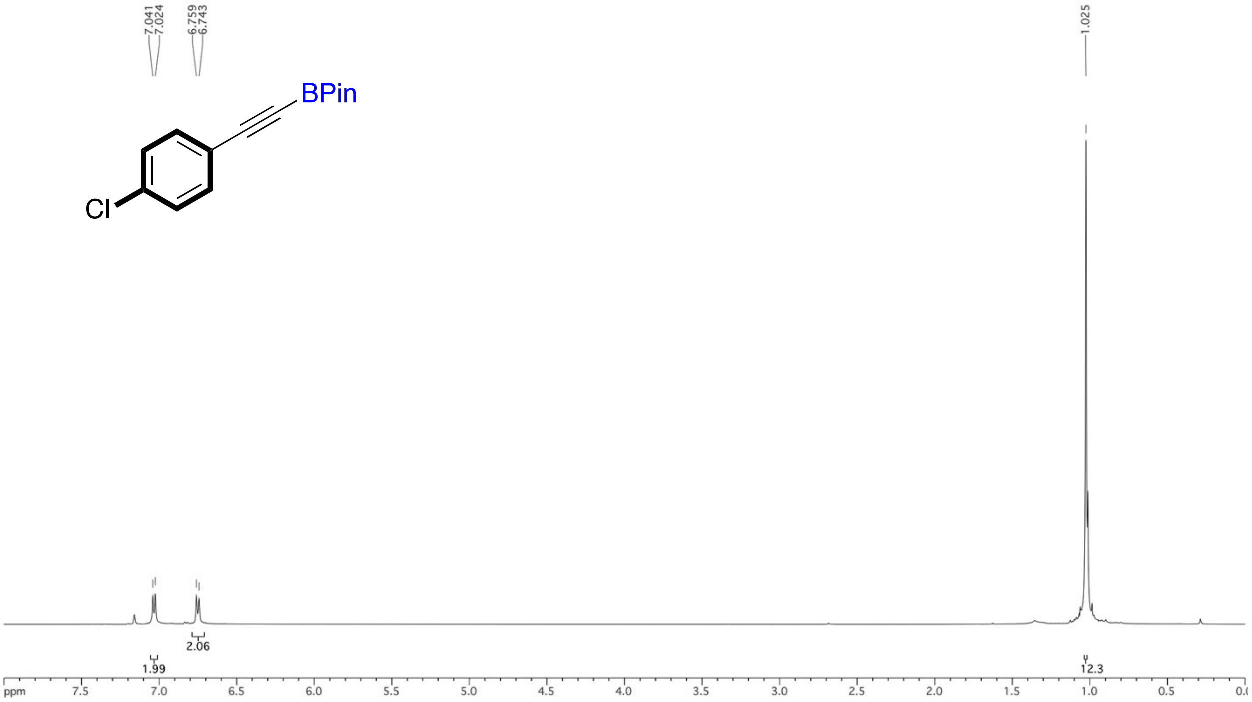
7.041
7.024
6.759
6.743
1.025
BPin
Cl
1.99
2.06
12.3
ppm 7.5 7.0 6.5 6.0 5.5 5.0 4.5 4.0 3.5 3.0 2.5 2.0 1.5 1.0 0.5

^{13}C

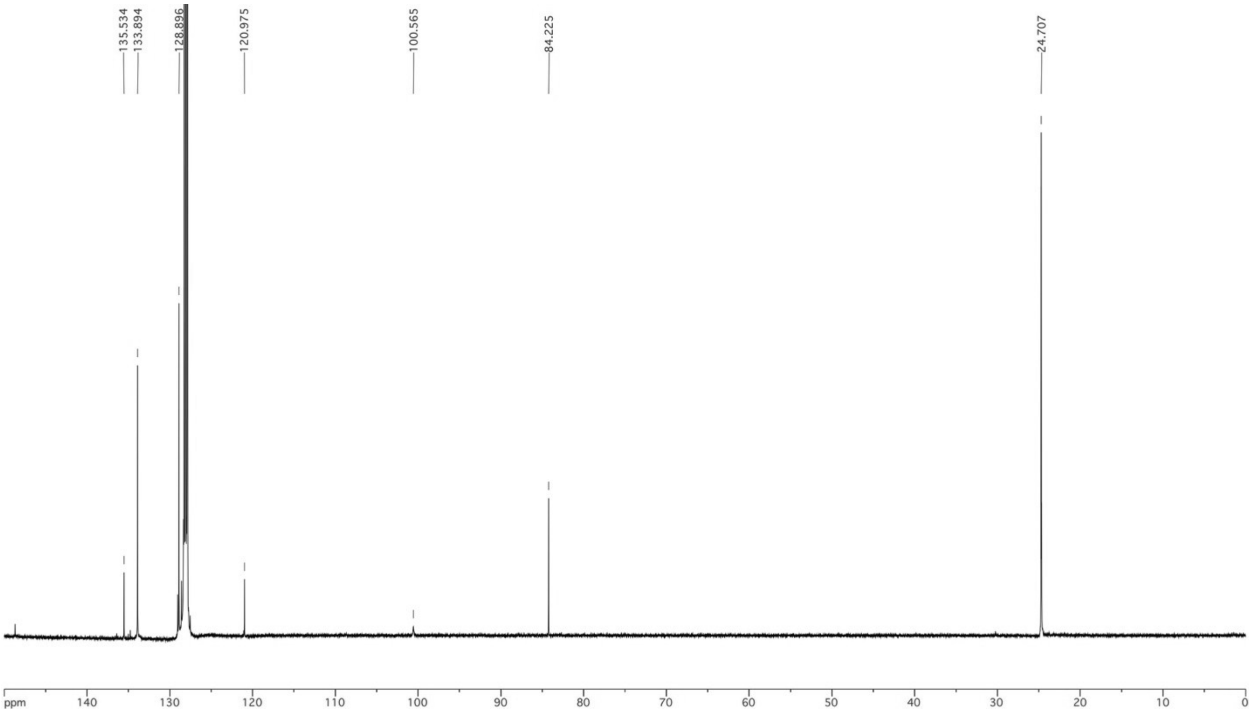
135.534
133.894
128.896
120.975
100.565
84.225
24.707
ppm 140 130 120 110 100 90 80 70 60 50 40 30 20 10 0

B_8

^{1}H

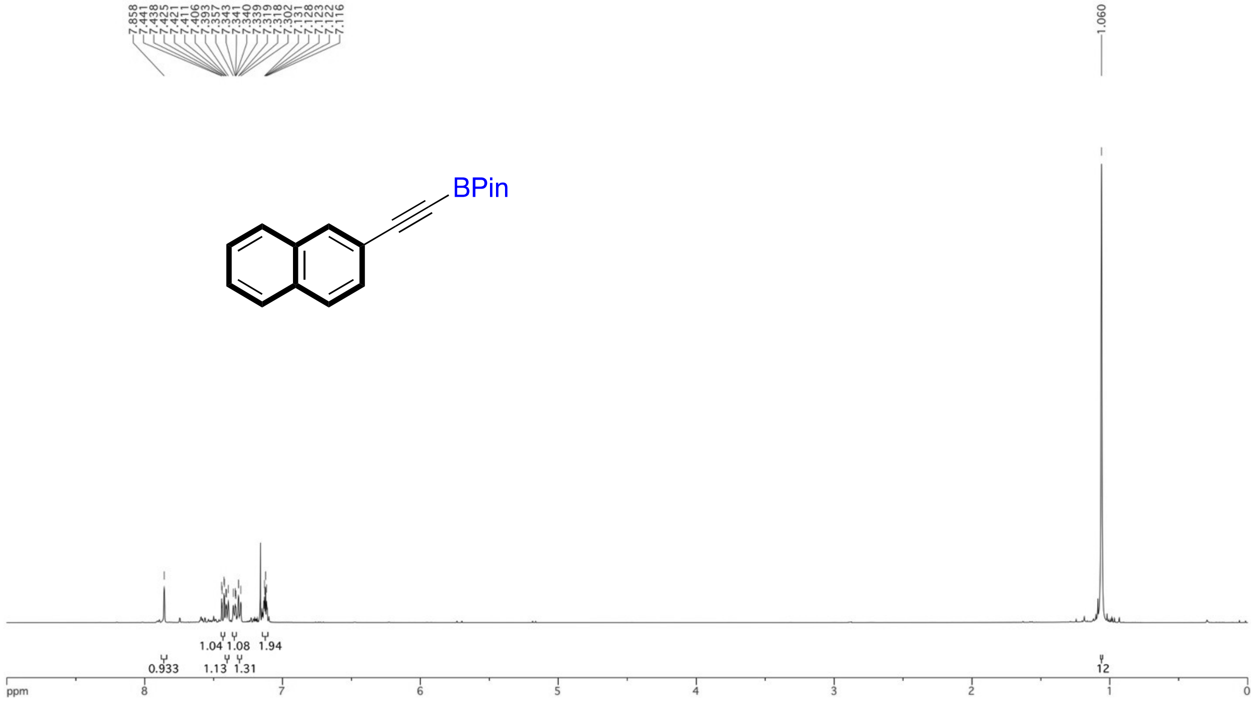
7.858
7.441
7.438
7.425
7.421
7.411
7.406
7.393
7.357
7.343
7.341
7.340
7.339
7.319
7.318
7.302
7.131
7.128
7.123
7.122
7.116
1.060
BPin
1.04
1.08
1.94
0.933
1.13
1.31
12
ppm
8
7
6
5
4
3
2
1
0

^{13}C

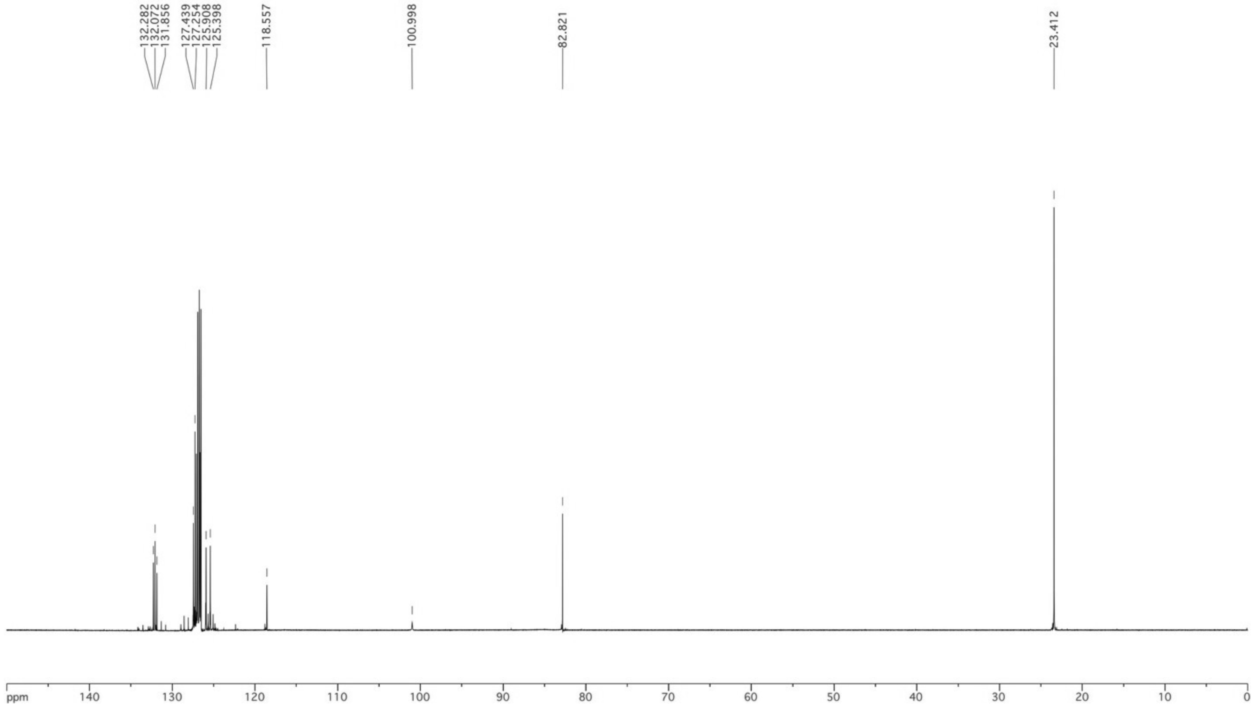
132.282
132.072
131.856
127.439
127.254
125.908
125.398
118.557
100.998
82.821
23.412
ppm
140
130
120
110
100
90
80
70
60
50
40
30
20
10
0

B_9

^{1}H

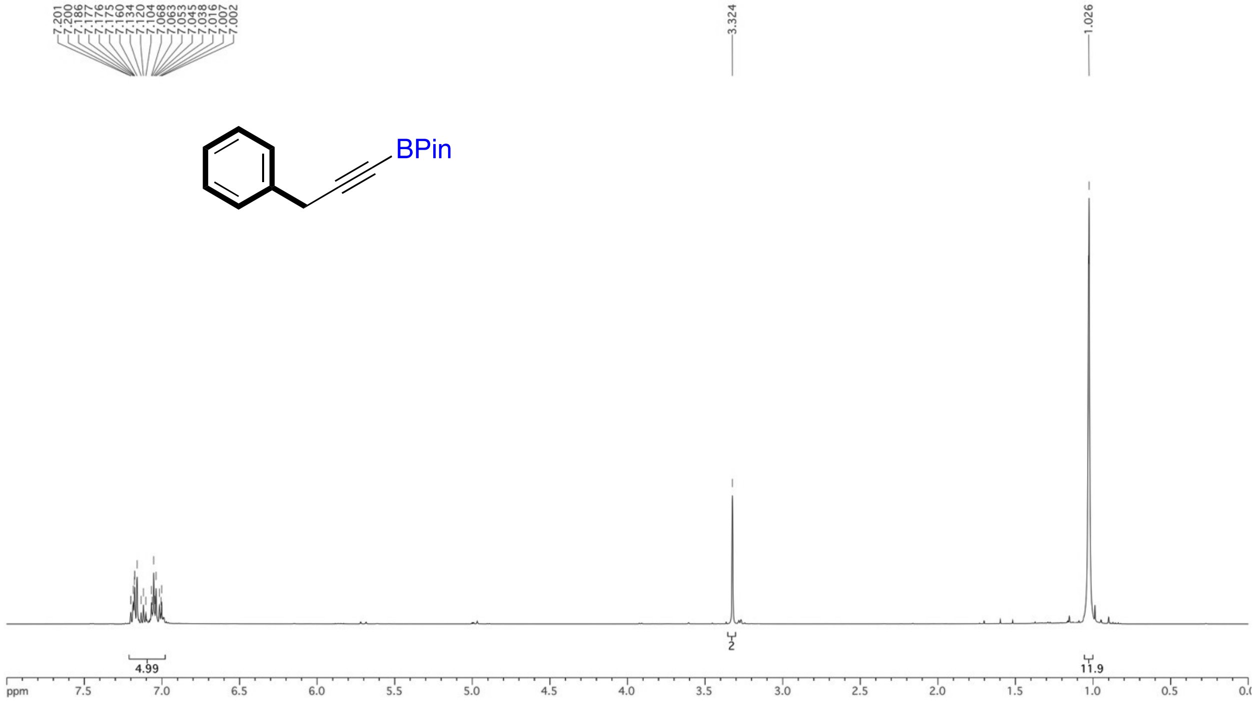
7.201
7.200
7.186
7.177
7.176
7.175
7.160
7.134
7.120
7.104
7.068
7.063
7.053
7.045
7.038
7.016
7.007
7.002
3.324
1.026
BPin
4.99
2
11.9
ppm

^{13}C

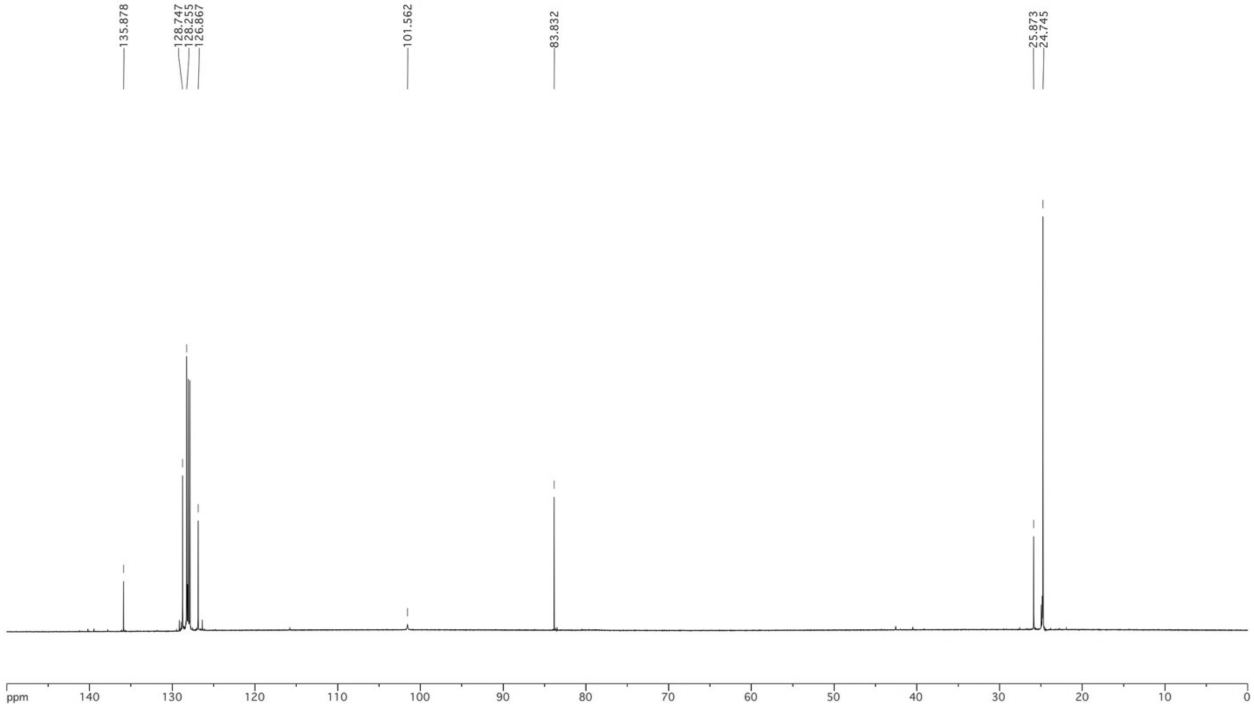
135.878
128.747
128.255
126.867
101.562
83.832
25.873
24.745
ppm

B_{14}

^{1}H

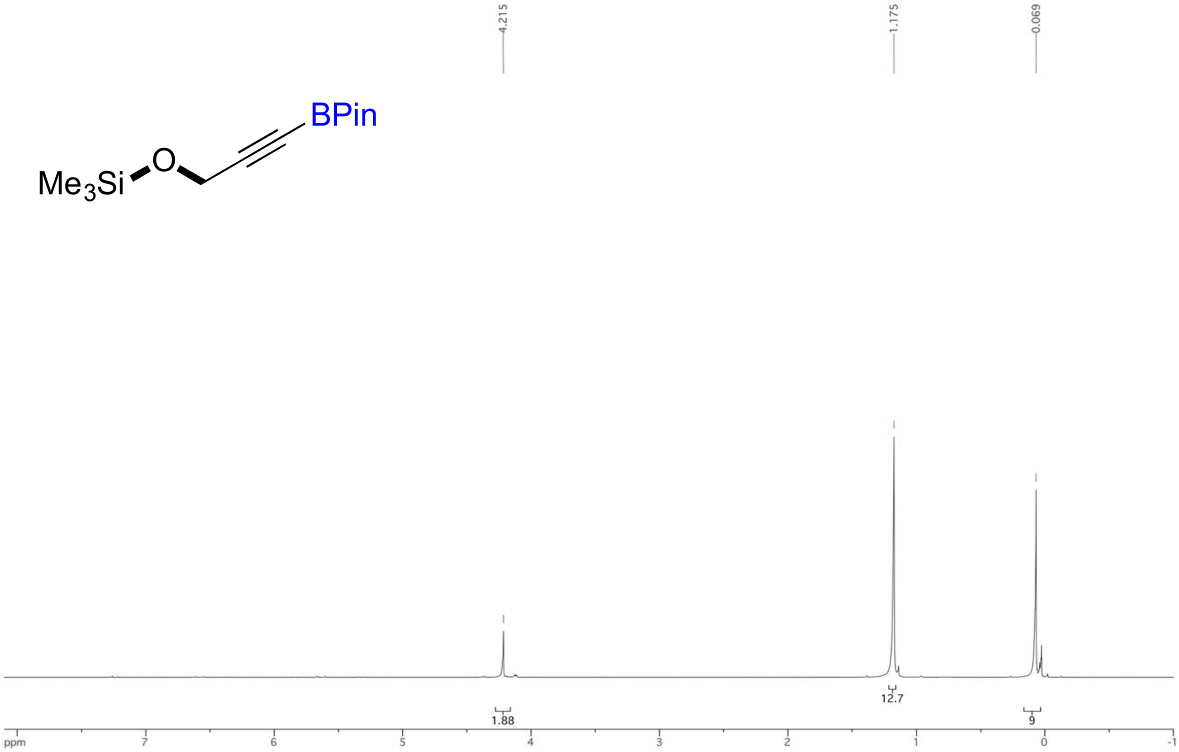
4.215
1.175
0.069
BPin
Me3Si
O
1.88
12.7
9
ppm

^{13}C

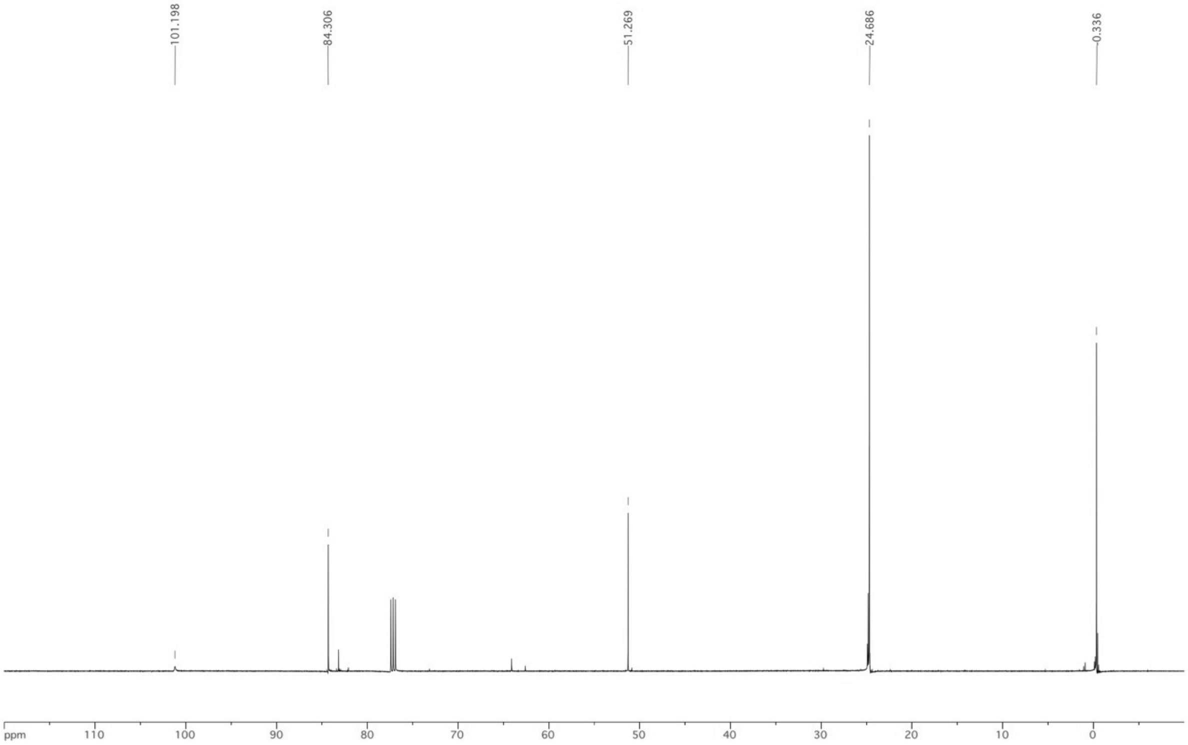
101.198
84.306
51.269
24.686
0.336
ppm

B_{11} Crude NMR

^{1}H

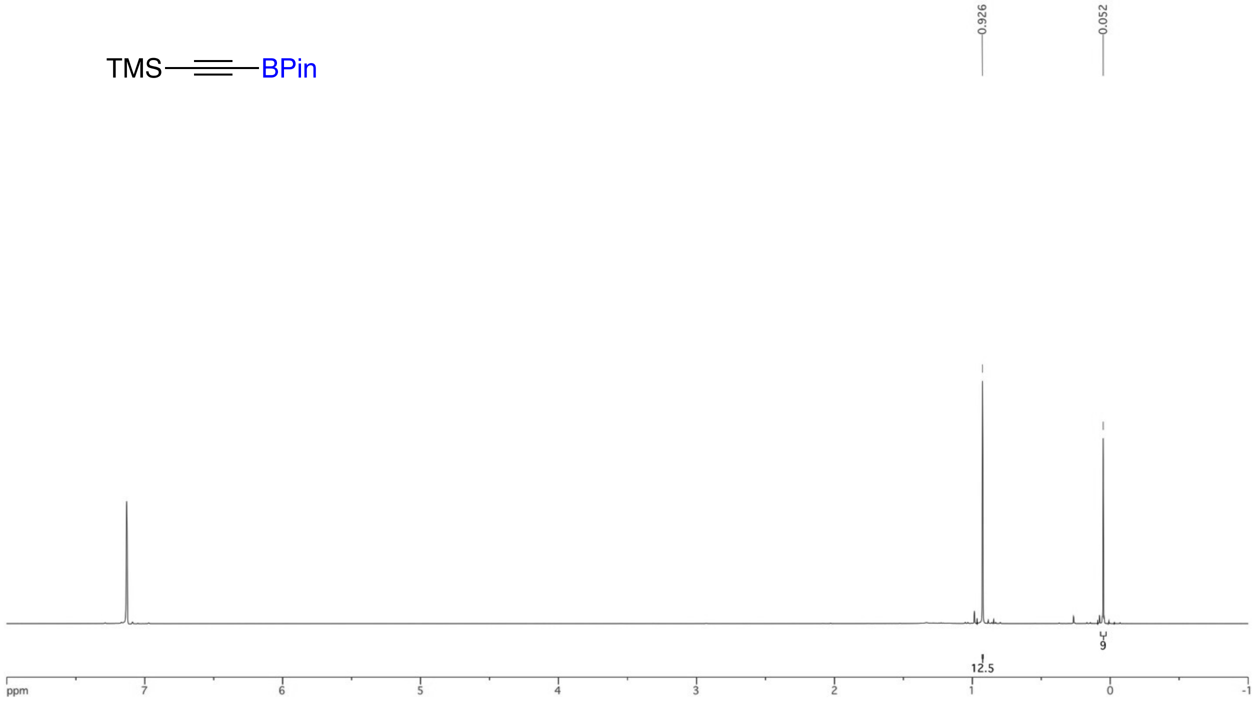

^{13}C

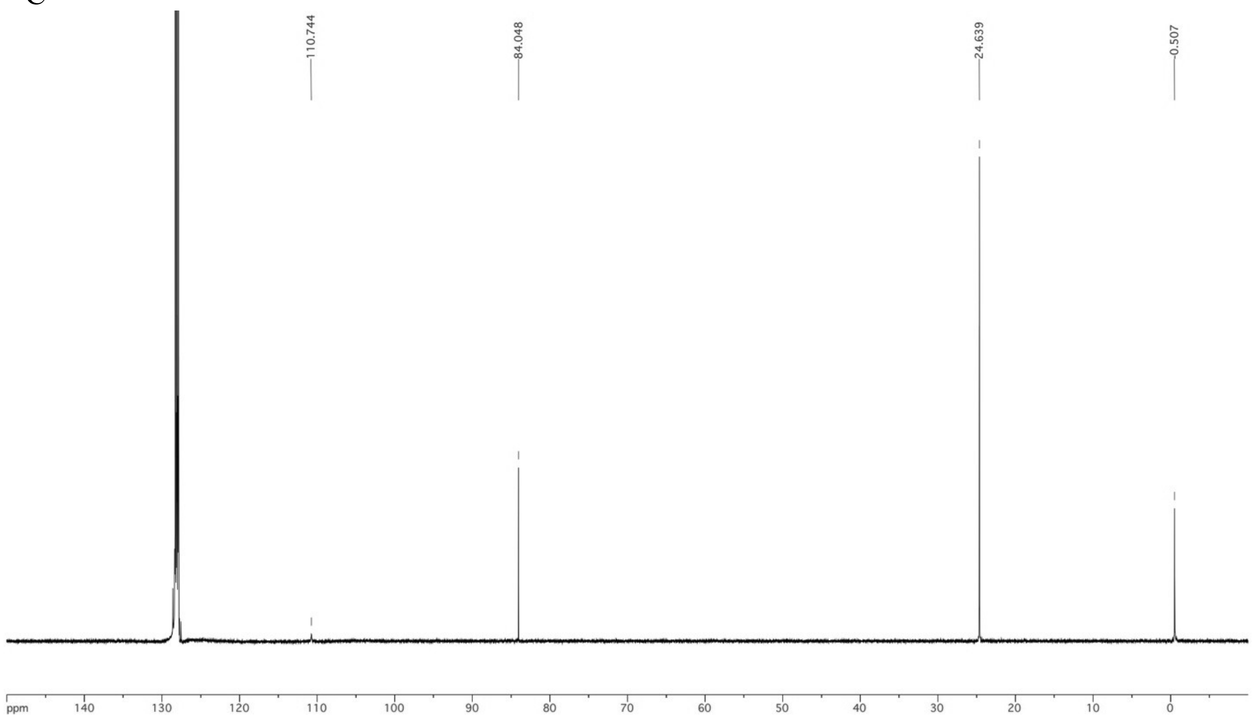

B_{12}

^{1}H

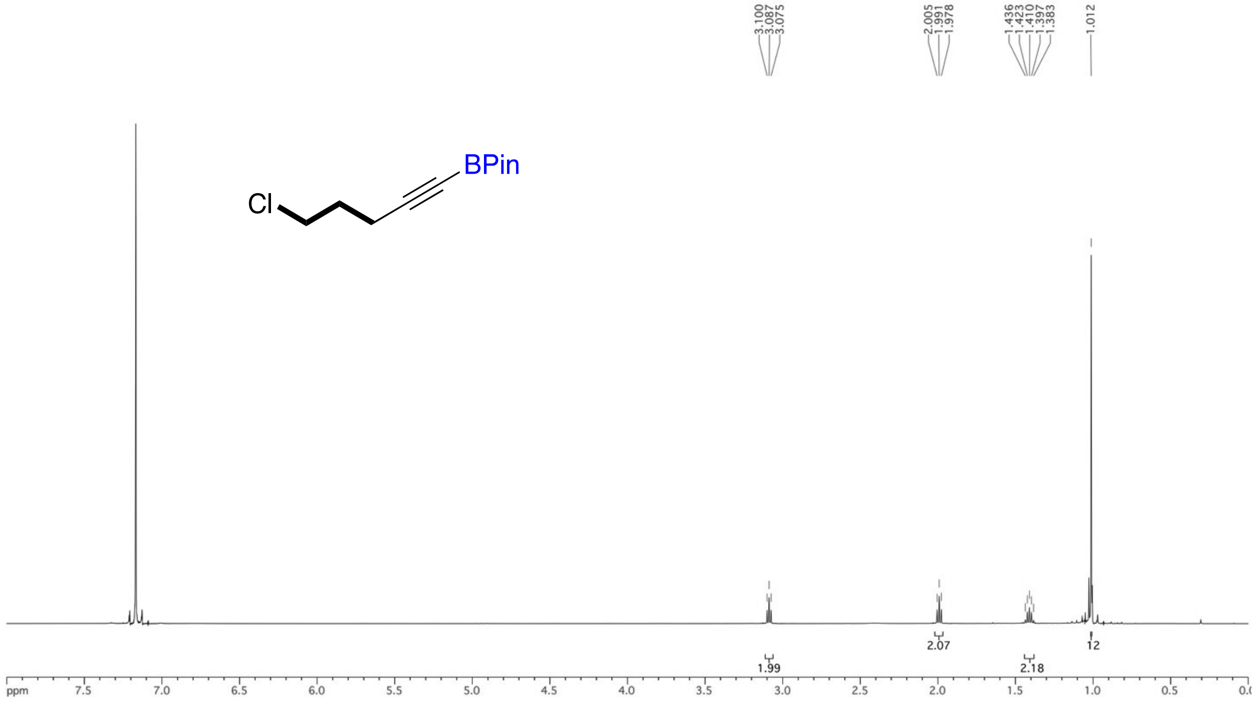
3.100
3.087
3.075
2.005
1.991
1.978
1.436
1.423
1.410
1.397
1.383
1.012
BPin
Cl
1.99
2.07
2.18
12
ppm

^{13}C

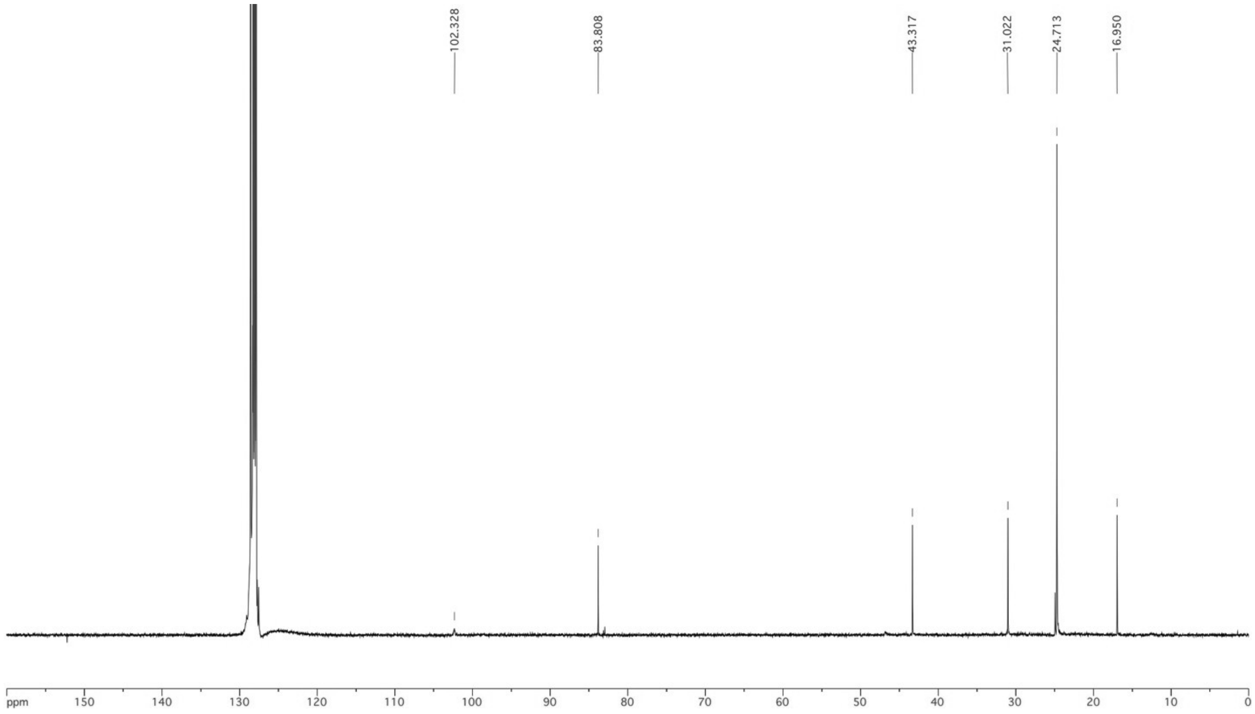
102.328
83.808
43.317
31.022
24.713
16.950
ppm

B_{13}

^{1}H

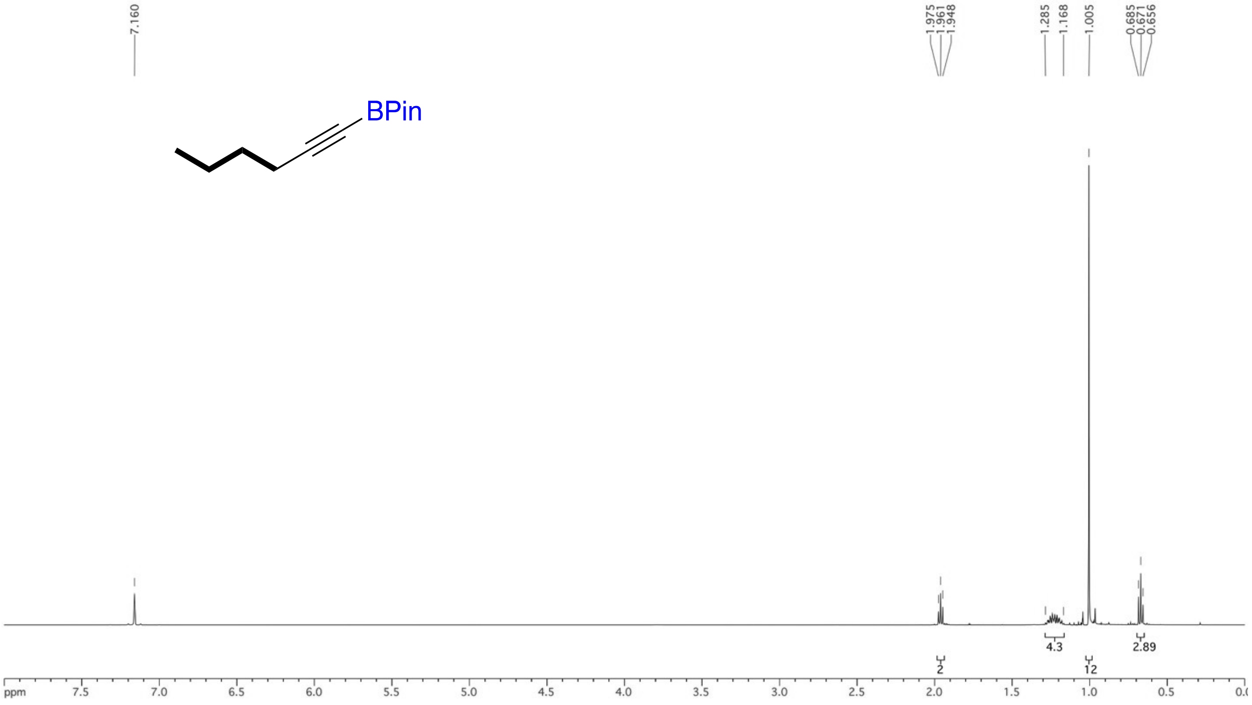
BPin
7.160
1.975
1.961
1.948
1.285
1.168
1.005
0.685
0.671
0.656
4.3
2.89
2
12
ppm 7.5 7.0 6.5 6.0 5.5 5.0 4.5 4.0 3.5 3.0 2.5 2.0 1.5 1.0 0.5

^{13}C

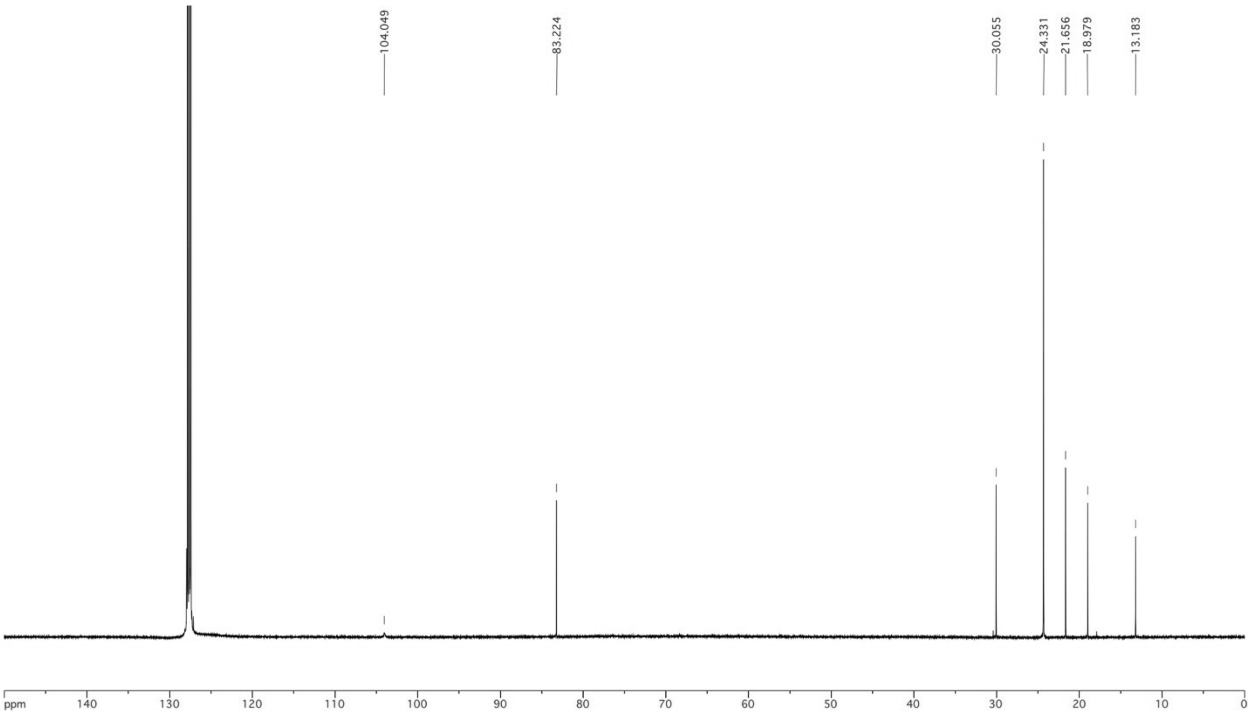
104.049
83.224
30.055
24.331
21.656
18.979
13.183
ppm 140 130 120 110 100 90 80 70 60 50 40 30 20 10 0

B_{14}

^{1}H

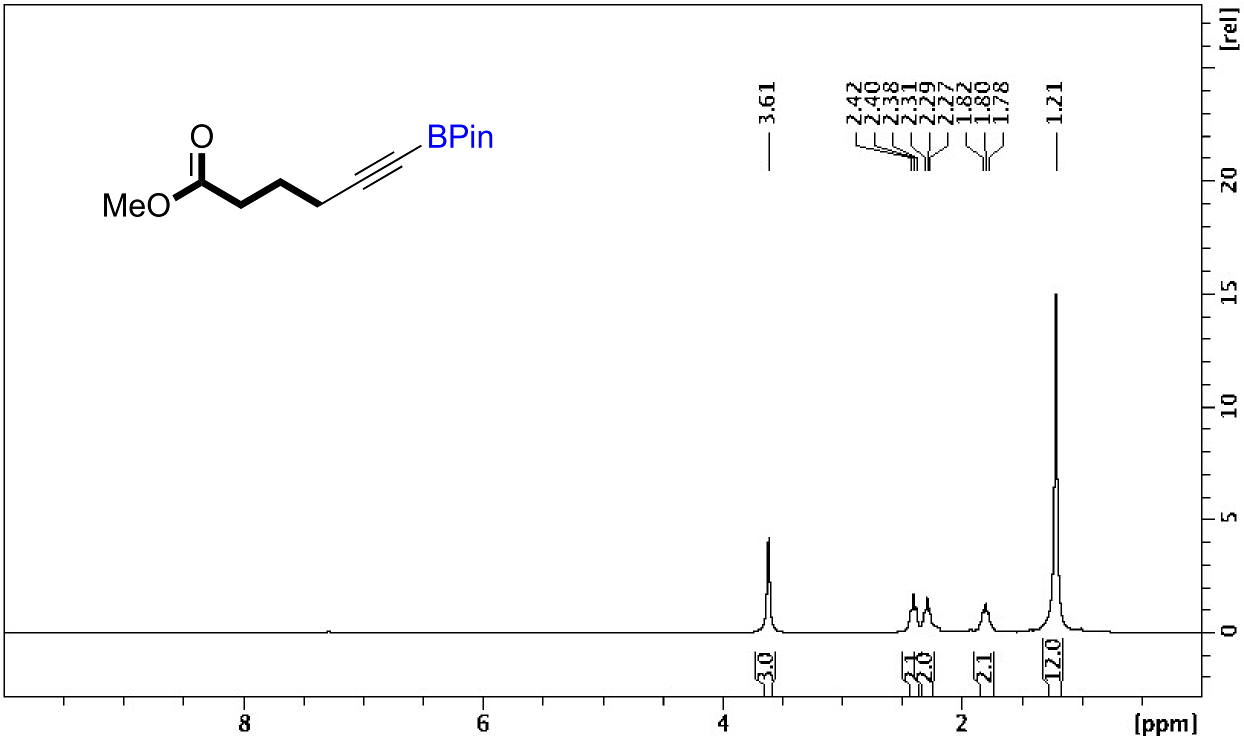
O
MeO
BPin
3.61
2.42
2.40
2.38
2.31
2.29
2.27
1.82
1.80
1.78
1.21
3.0
2.1
2.0
2.1
12.0
[rel]
[ppm]

^{13}C

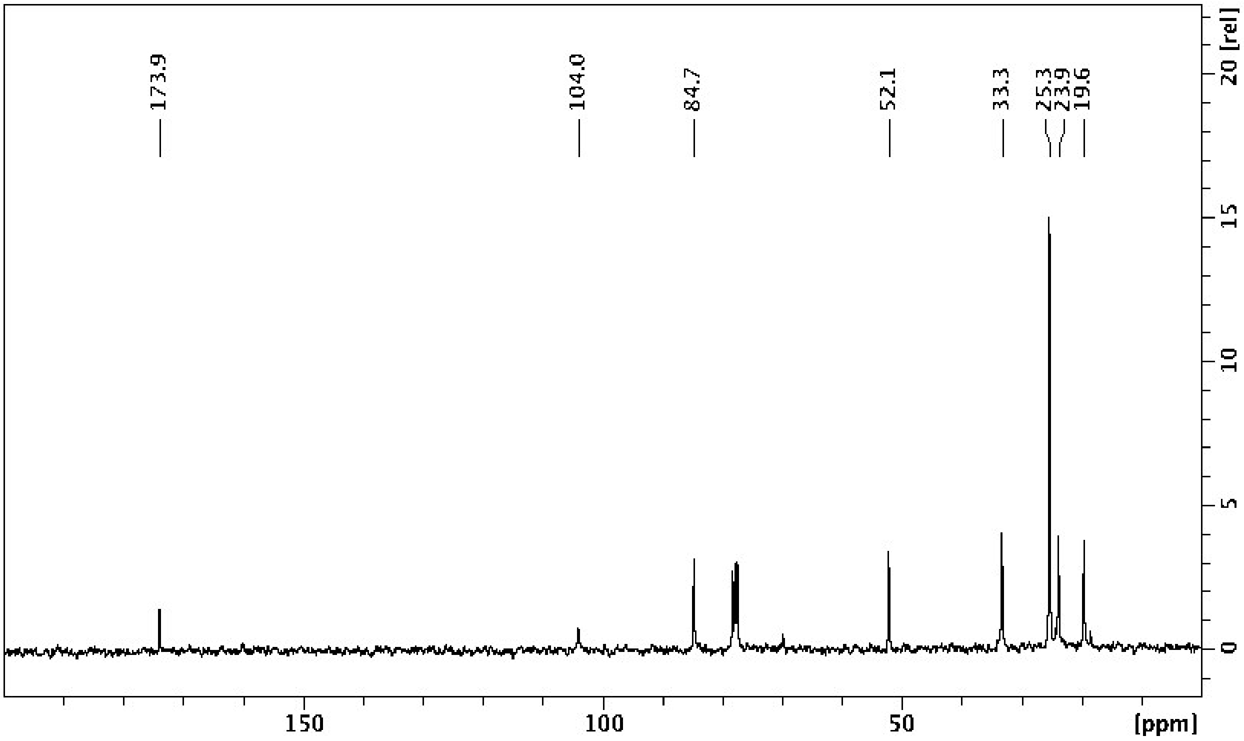
173.9
104.0
84.7
52.1
33.3
25.3
23.9
19.6
[rel]
[ppm]

B_{15}

^{1}H

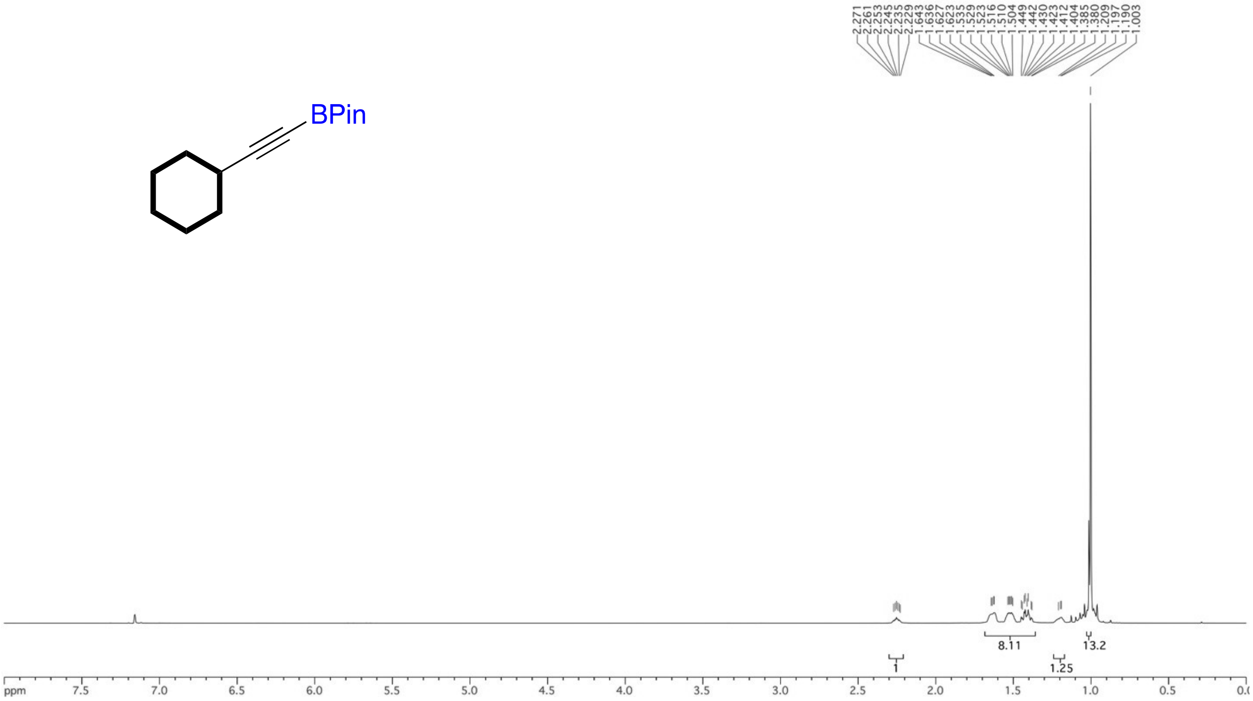
BPin
2.271
2.261
2.253
2.245
2.235
2.229
1.643
1.636
1.627
1.623
1.535
1.529
1.523
1.516
1.510
1.504
1.449
1.442
1.430
1.423
1.412
1.404
1.385
1.380
1.209
1.197
1.190
1.003
8.11
13.2
1
1.25
ppm 7.5 7.0 6.5 6.0 5.5 5.0 4.5 4.0 3.5 3.0 2.5 2.0 1.5 1.0 0.5

^{13}C

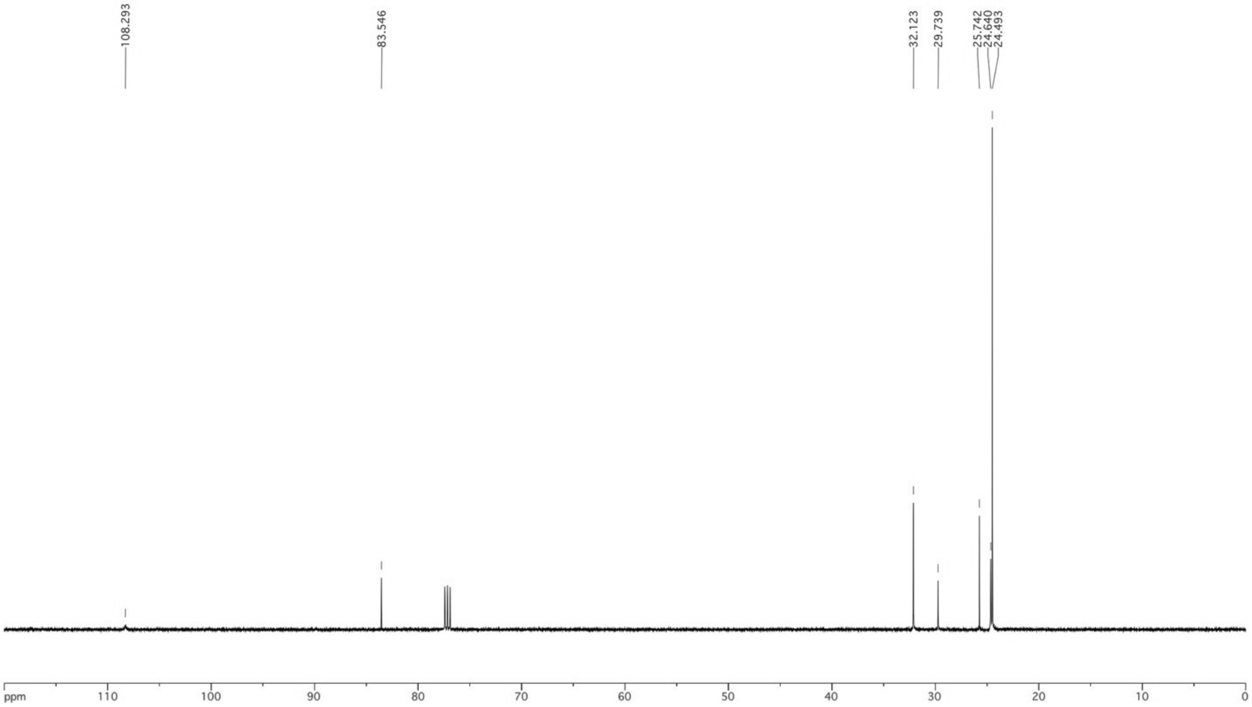
108.293
83.546
32.123
29.739
25.742
24.640
24.493
ppm 110 100 90 80 70 60 50 40 30 20 10 0

[i] L. Jin, D. R. Tolentino, M. Melaimi, G. Bertrand. *Sci. Adv.* **2015**, *1,* e1500304.

[ii] L. Cheng and C. J. Cordier, *Angew. Chem. Int. Ed.* 2015, **54**, 13734.

[iii] H. E. Ho, N. Asao, Y. Yamamoto, T. Jin. *Org. Lett.*, **2014**, 16, 4670.

[iv] S. I. Gazic, E. Casas-Arce, S. J. Roseblade, U. Nettekoven, A. Zanotti-Gerosa, M. Kovacevic, Z. Casar. *Angew. Chem. Int. Ed.* **2012**, *51*, 1014.

[v] C. I. Lee, J. C. DeMott, C. J. Pell, A. Christopher, J. Zhou, N. Bhuvanesh, O. V. Ozerov, *Chem. Sci.* **2015,** *6*, 6572

[vi] G. A. Molander, N. M. Ellis. *J. Org. Chem.* **2008**, *73*, 6841.

[vii] Y. Nishihara, Y. Okada, J. Jiao, M. Suetsugu, M.-T. Lan, M. Kinoshita, M. Iwazaki, K. Takagi. *Angew. Chem. Int. Ed.* **2011**, *50*, 8660.

[viii] A. Di-Giuseppe, R. Castarlenas, J. J. Pérez-Torrente, F. J., Lahoz, V. Polo, L. A. Oro *Angew. Chem. Int. Ed.*, **2010**, *50*, 3938.
